# The transcriptome of early compensatory kidney growth reveals cell and time specific responses

**DOI:** 10.1016/j.isci.2024.110608

**Published:** 2024-07-27

**Authors:** Darling M. Rojas-Canales, Soon Wei Wong, Elise J. Tucker, Anthony O. Fedele, Kym McNicholas, Anne-Sophie Mehdorn, Jonathan M. Gleadle

**Affiliations:** 1 Department of Renal Medicine, Southern Adelaide Local Health Network, Flinders Medical Centre, Bedford Park, SA, Australia; 2 Flinders University, College of Medicine and Public Health, Flinders Health and Medical Research Institute, Adelaide, SA, Australia; 3 Department of General, Abdominal, Thoracic, Transplantation and Paediatric Surgery, University Hospital Schleswig-Holstein, Campus Kiel, Kiel, Germany

**Keywords:** Nephrology, Morphologic abnormality, Integrative aspects of cell biology, Transcriptomics

## Abstract

Following kidney removal, the remaining kidney enlarges and increases its function. The mechanism and signals driving this compensatory kidney hypertrophy and the enlargement of its constituent kidney cells remains elusive. RNA-seq studies in mice undergoing hypertrophy 24, 48, and 72 h following nephrectomy were undertaken to understand the early transcriptional changes. This revealed substantial enhancement of cholesterol biosynthesis pathways, increases in mitochondrial gene expression and cell cycle perturbations. Single nuclei RNA-seq delineated cell specific changes at 24 h post nephrectomy and showed that sterol binding protein 2 (SREBP2) activity increases in medullary thick ascending limb cells in keeping with promotion of cholesterol synthesis. Cultured renal tubular cells were examined for insulin-like growth factor-1 (IGF-1) stimulated hypertrophy and SREBP2 was found to be required for increase in cell size. This work describes the early cell specific growth pathways mediating cellular and kidney hypertrophy with an intriguing role for cholesterol synthesis.

## Highlights

• Cholesterol, mitochondria, and cell cycle genes altered in hypertrophying kidneys
• Cholesterol biosynthesis gene expression is key hallmark at 24 h in remaining kidney
• Single nuclei RNA-seq reveals specific proximal tubular cell changes
• IGF-1 induced renal cell hypertrophy involves SREBP2 in size increase

## Graphical abstract

**Figure:**
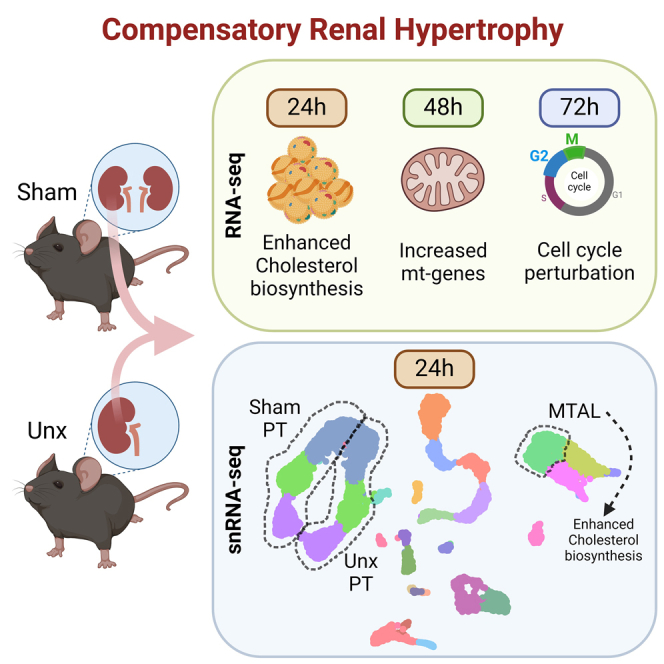

## Introduction

Compensatory organ growth is a localized physiological response to organ mass loss following removal of the organ. Many bilateral organs have this ability including the kidney, where growth is dominated by hypertrophy, and thus enlargement of individual kidney cells.^1^ When a kidney is removed for organ donation, the remaining kidney usually compensates for the nephrectomy by growing in volume and increasing in function.^1^ Similar compensatory hypertrophy can be seen in the remaining kidney in patients undergoing nephrectomy for kidney cancer^2^ and in transplanted kidneys^3^ and are often associated with long term enhancements of kidney function. This contrasts with the deleterious effects of hyperfiltration that are often seen with more substantial experimental loss of renal mass (e.g., 5/6th nephrectomy) or with other causes of pathological hypertrophy such as diabetic nephropathy.^4^ The mechanisms driving the remaining kidney to naturally enlarge and increase its function remain elusive.^1^ Understanding the mechanism driving compensatory renal hypertrophy will provide fundamental insights into growth control of cells and organs and a potential route for targeted therapies that could enhance kidney size and function.

The process of hypertrophy is rapid and occurs within days, in humans, studies have shown a 21% increase in renal parenchymal volume and GFR (glomerular filtration rate) within the first 3 days and 2 days respectively, with well-established hypertrophic growth at 1 month post nephrectomy. In rodents the response appears to be accelerated with hypertrophic growth evident with the first 2 days post nephrectomy.^1^ However, the mechanism by which the loss of the other kidney is sensed is unclear and the initial trigger to growth has not been identified. The growth increase is restricted to the contralateral kidney and circulating mediators have been suggested.^5^^,^^6^^,^^7^ The response of kidneys post-nephrectomy is primarily reflected by proximal tubular cell hypertrophy. Several strands of evidence have suggested that renal cell hypertrophy is mediated through cell cycle alterations,^8^ with tubular cells transitioning from quiescence and arresting in a growth phase, leading to increased cell size and protein synthesis mediated by mTOR (mammalian target of rapamycin) signaling.^9^^,^^10^ However, the stimulus, signaling and transcriptional control of such a response has not been fully defined. Growth factors, notably insulin-like growth factor, have been implicated in the stimulation of compensatory renal hypertrophy,^11^^,^^12^^,^^13^^,^^14^^,^^15^^,^^16^ but again an understanding of the mechanisms of induction, the cellular sources and restriction of the growth response to the remaining kidney is lacking.

Only limited global transcriptional profiling studies and no single cell studies have been reported to examine the molecular events in compensatory renal hypertrophy.^17^^,^^18^^,^^19^ This paper addresses this by providing insights into the early transcriptional events via both bulk and single nuclei RNA-seq transcriptional profiling of murine kidneys in the initial stages of hypertrophy. The results reveal cell cycle perturbation, enhancement of mitochondrial gene expression and for the first time demonstrate modulation of cholesterol biosynthesis gene expression. Sterol regulatory element-binding protein 2 (SREBP2), a primary driver of cholesterol biosynthesis activated by both IGF-1^20^ and mTORC1^21^ is shown to have a role in driving growth *in vitro*. Single nuclei RNA-seq analysis shows that changes in expression of genes required for cholesterol synthesis are cell type specific.

## Results

### Differential gene expression of remaining kidneys in nephrectomised versus sham operated mice

**Figure 1:**
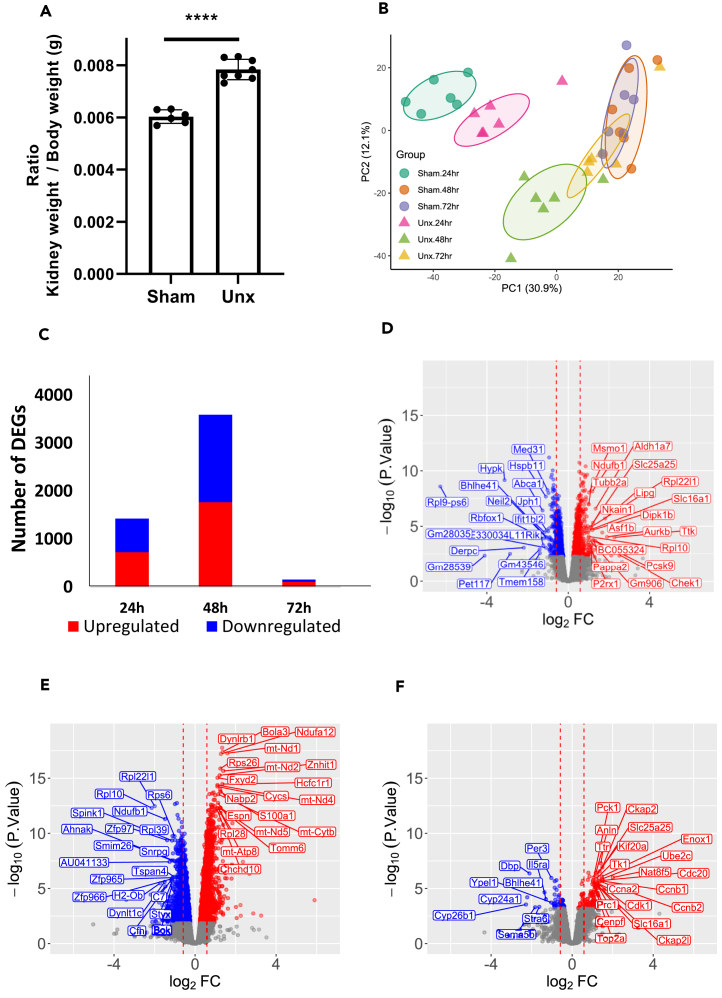
RNA-seq analysis during the early stages of compensatory renal hypertrophy following a unilateral nephrectomy Mice (C57BL6) underwent sham or left unilateral nephrectomy surgery and at 24, 48, and 72 h following surgery, the remaining kidney was harvested, RNA extracted, and RNA sequencing undertaken. At the 2-week time point, kidneys were collected and weighed as a measure of renal hypertrophy. (A) Ratio of kidney weight to body weight, 2 weeks following a left unilateral nephrectomy (*n* = 8) or sham operation (*n* = 6) ± SEM ∗∗∗∗*p* < 0.0001. (B) Principal-component analysis (PCA) of gene expression in the individual samples. The ellipse assumes a multivariate t-distribution of the sample groups. (C) Bar graph showing DEGs at 24, 48, and 72 h post-nephrectomy surgery when compared to sham surgery at the same time points. Red depicts upregulated genes and blue shows down regulated genes. Volcano plots displaying significant (adjusted *p* value <0.05) differentially expressed genes between Unx (nephrectomy) and sham surgery at (D) 24 h, (E) 48 h, and (F) 72 h post-surgery. The y axis corresponds to the –log_10_ (*p* value), the x axis displays the log_2_ fold change value, and the red vertical dotted lines indicate a log_2_ fold change of 0.5. The red dots represent the upregulated genes and the blue dots represent genes whose expression is downregulated in Unx. The top 20 upregulated and top 20 downregulated genes with log_2_ fold change of >1 or < −1 are labeled. See also Figure S1 and Tables S1–S6.

Removal of the left kidney resulted in compensatory hypertrophy of the remaining kidney with a 27% increase in kidney to body weight ratio after 2 weeks (Figure 1A), which was accompanied with an increase in kidney size (See Figure S1A). In separate experiments, the remaining right kidney was collected at 24, 48, and 72 h (*n* = 6 at each time point) following sham surgery or uninephrectomy and investigated by bulk RNA-seq. Principal-component analysis (PCA) of the RNA-seq data revealed a tendency of time points and the sham and Unx groups to cluster together (Figure 1B). All time points (24, 48, and 72 h) showed significant differential gene expression changes. The greatest number being evident 48 h post-nephrectomy followed by the number of differentially expressed genes (DEGs) at 24 h, with the least DEGs seen at 72 h (Figure 1C). The 20 most upregulated and 20 most downregulated genes (with log_2_ fold change of >1 or < −1 and adjusted *p* value <0.05) differentially expressed between Unx (nephrectomy) and Sham surgery at 24, 48, and 72 h post-surgery are shown in Figures 1D, 1E, 1F, and Tables S1–S6.

### Cholesterol biosynthesis and uptake genes are highly enriched at 24 h following nephrectomy

**Figure 2:**
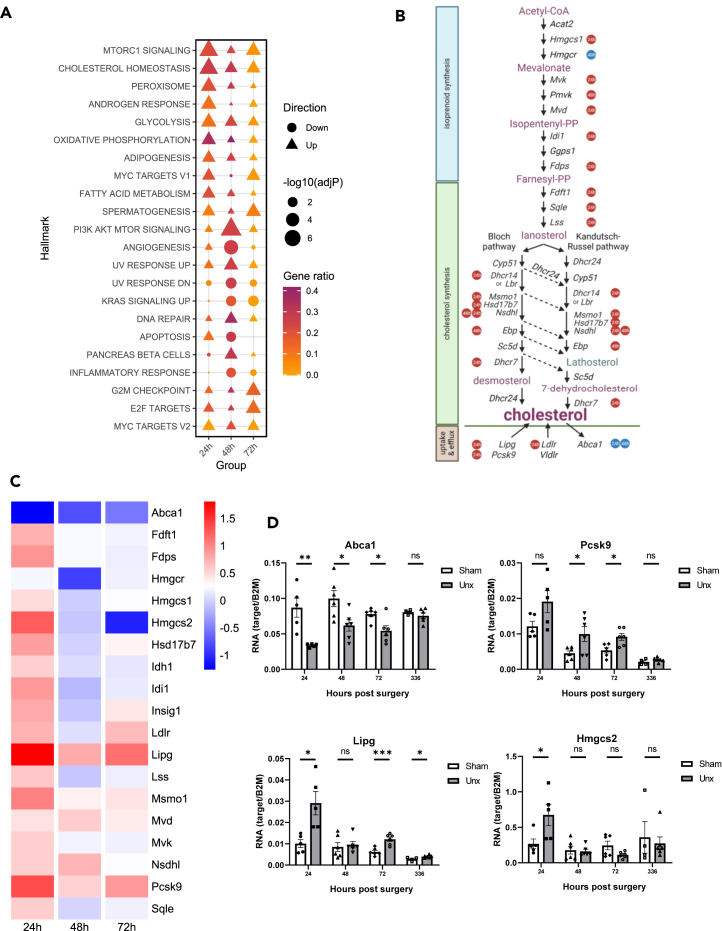
Hallmark Analysis of differential gene expression shows an early upregulation of cholesterol biosynthesis and uptake genes unique to 24 h following nephrectomy (A) Hallmark gene set enrichment testing. Enriched gene sets were filtered for FDR <0.01 and combined for plotting using a ranked list from each group with the top 10 most significant (FDR <0.01) shown. Gene ratio is the differentially expressed genes from the group that overlapped with the hallmark gene set divided by the total number of genes in the gene set. Direction (down or up) indicates whether a gene set is positively enriched or negatively enriched in the group. (B) Schematic of the genes involved in cholesterol uptake, synthesis and efflux. Cholesterol can be synthesized via either the Kandutsch-Russell pathway (right) or the bloch pathway (left). Circular symbols labeled with 24 or 48 h next to each gene indicate whether it has significantly increased (red) or decreased (blue) at the respective time point post-nephrectomy. (C) Heatmap of genes with roles in cholesterol biosynthesis and transport across all timepoints. Differential expression of selected genes with roles in cholesterol biosynthesis, uptake, or efflux between Sham and uninephrectomy in the remaining kidney at 24, 48, and 72 h which showed statistically significant differential expression *p* ≤ 0.05 (top) or a log_2_foldchange of >1 (bottom). Genes which are downregulated are indicated in blue and upregulated in red. (D) qPCR mRNA expression in kidneys from sham and uninephrectomised mice 24–336 h post-surgery (n = 4–6, *p* < 0.05 ∗, *p* < 0.01∗∗, *p* < 0.001∗∗∗; ns = not significant). See also Figures S2 and S3.

Hallmark gene set analysis of all DEGs was performed. This revealed mammalian target of rapamycin complex 1 (mTORC1) signaling was the most significantly enriched gene set at 24 h following nephrectomy, with significant enrichment also seen at 48 and 72 h, as well as an enrichment of the overlapping PI3K/AKT/mTOR gene set at 48 h (Figure 2). This was consistent with prior work^22^^,^^23^ and immunoblotting showing an increase in ribosomal protein S6 (RPS6) phosphorylation (RPS6p-s240/244) at 24 h (Figure S1).

Intriguingly, the next most significantly enriched hallmark gene set was cholesterol homeostasis, showing significant enrichment at all three time points (Figure 2). Having identified cholesterol homeostasis as a process not previously identified with a role in CRH, analysis was extended to all genes with roles in cholesterol biosynthesis. As illustrated in the schematic of cholesterol metabolism in (Figure 2B) heatmap (Figure 2C), showing nearly all genes involved in cholesterol biosynthesis (e.g., *Pcsk9*, *Fdft1*, *Fdps*, *Idh1*, *Idi1*, *Insig1*, *Lss*, *Msmo1*, *Nsdhl*, and *Sqle*) were significantly induced at 24 h, while genes involved in cholesterol uptake (*Lipg* and *Ldlr*) also showed increased expression and a gene with roles in cholesterol efflux showed repression (*Abca1*). This distinct gene pattern was most prominent at the earliest (24 h) time point (see Figure S2).

To provide independent validation of this gene regulation, qPCR assays confirmed induction of *Pcsk9*, *Lipg*, and repression of *Abca1* (Figure 2D), with similar patterns of differential expression between the RNA-seq and qPCR. This pattern of gene regulation with broad enhancement of genes with roles in cholesterol biosynthesis (but not triglycerides) and uptake suggests promotion of cholesterol biosynthesis and uptake and induction by sterol regulatory element binding protein 2 (SREBP2). There was also a significant increase in a mevalonate pathway related gene *Hmgcs2* 24 h post-nephrectomy, which is localized in the mitochondria and known to drive ketogenic responses.^24^

Untargeted mass spectrometric analysis 24 h post-surgery (Figure S3) was undertaken to examine correlations of peptide alterations with RNA-seq. Protein and RNA integration analysis showed 13 commonly upregulated proteins and mRNAs. Hallmark enrichment analysis showed significant increases in pathways of cholesterol homeostasis, fatty acid metabolism, and mTORC1 signaling (Figure S3D).

### Mitochondrial transcripts are enhanced 48 h following nephrectomy

**Figure 3:**
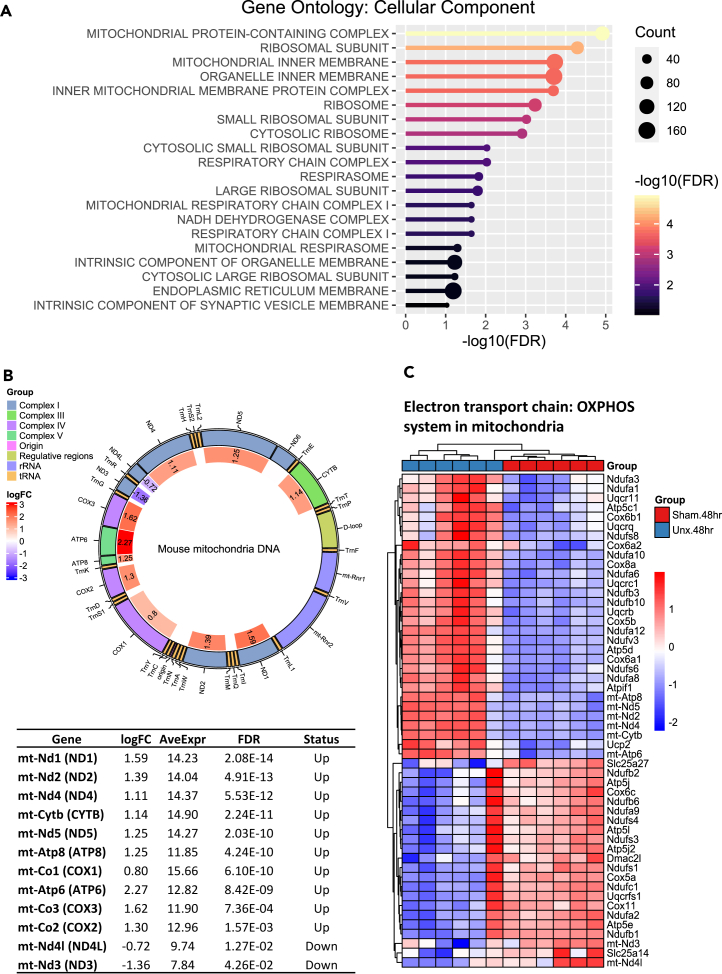
Regulation of mitochondrially encoded transcripts at 48 h following nephrectomy (A) Gene ontology (cellular component) analysis performed using Enrichr with genes that were significantly regulated (FDR <0.05). (B) Schematic of the mouse mitochondrial genome showing different groups of mitochondrially encoded transcripts that were significant differently regulated (FDR <0.05) in the unx vs. sham kidneys at 48 h. The table below indicates the gene name, log_2_FC, average level of expression, FDR and direction of regulation from the RNA-seq data. (C) Heatmap of z-scaled logCPM of genes involved in electron transport chain—Oxidative phosphorylation in the mitochondria.

A gene ontology enrichment analysis using the cellular component category and demonstrated significant enrichment of genes in mitochondrial components (Figure 3A). Gene transcripts encoded by the mitochondrial genome showed significant and co-ordinated increased expression in the remaining kidneys of the nephrectomized animals versus sham surgery (Figure 3B). These genes included *Nd1*, *Nd2*, *Nd4*, *Cytb*, *Cox1*, *Cox2*, *Cox3*, *Atp6*, and *Atp8* with induction ranging from log_2_FC of 0.8–2.27 and with FDRs (false discovery rate) from 1.5 × 10^−3^ – 2.1 × 10^−14^. As shown in Figure 3B, the other mitochondrial transcripts showed no significant regulation, though two adjacent transcripts (*Nd4l* and *Nd3*) did show reduction in expression, albeit with much less statistical certainty. Furthermore, there was also significant upregulation of nuclear encoded genes with mitochondrial functions such as *Ndufa12*, *Cycs*, *Tomm6*, *Cox6a2*, and *Hmgcs2*. Such induction was most prominent at 48 h with less or no significant induction of mitochondrial transcripts at 24- and 72-h post-nephrectomy. Additionally, hallmark gene set analysis (Figure 2A) showed significant enhancement of oxidative phosphorylation at 24, 48, and 72 h. Genes involved in the oxidative phosphorylation system in the mitochondria at 48 h post-nephrectomy (Figure 3C), showed a significant number of genes that were up and down regulated at this time point.

### Genes involved in cell cycle control are enhanced at 72 h following nephrectomy

**Figure 4:**
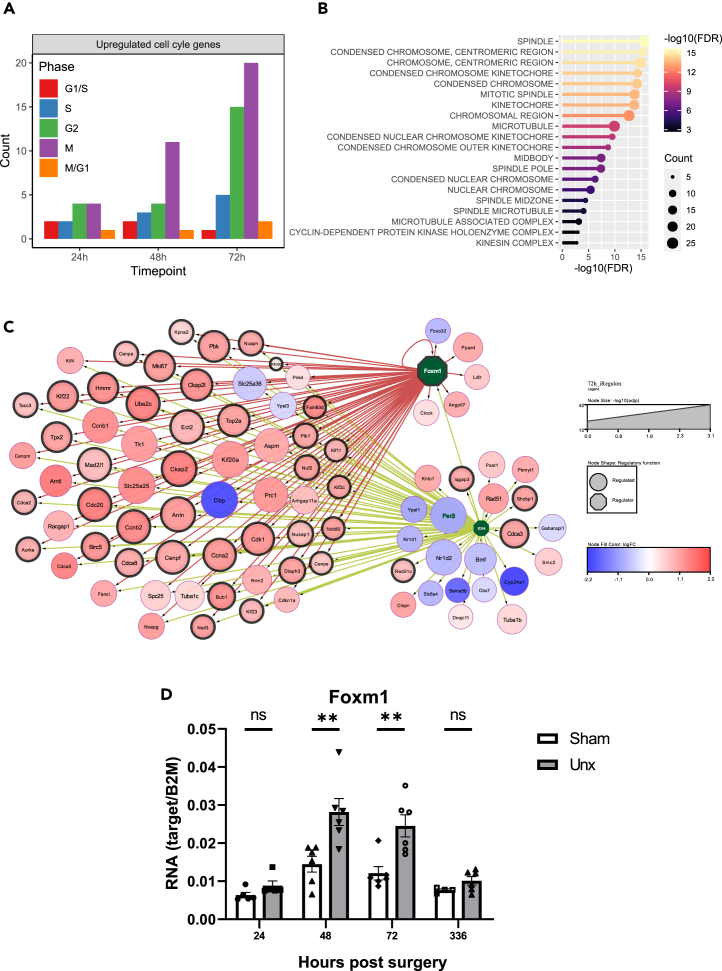
Regulation of genes with roles in cell cycle control at 72 h following nephrectomy Cell cycle gene sets from Hsiao et al. (2020) were used to perform Fry enrichment analysis for all timepoints (24, 48, and 72 h). (A) Bar plot showing the number of DE genes with FDR <0.05 and logFC >1 overlapping with genes in each cell cycle phases as characterized by Hsiao et al. (2020). (B) Gene ontology (GO) enrichment analysis at 72 h. Top 20 most significantly enriched GO terms (−log_10_ (*p-*value)) of the target genes in the cellular components. (C) Upstream regulon analysis of the genes differentially regulated between unx and sham operated mice at 72 h. Cytoscape add-in iRegulon analysis to determine the enrichment of transcription factor-binding sites in close proximity (20 kb centered around TSS) to the gene promoter which showed significant (FDR <0.05) regulation at 72 h post nephrectomy. Transcription factors with normalized enrichment score >9 and FDR on motif similarity <0.001 were selected for network plot. Downregulated genes in nephrectomised mice are indicated blue and up-regulated in red. Regulators are denoted by the hexagonal shape and regulated genes by the circle. The size of the circle indicates the -log_10_(adjp) and a black border indicates genes with increased expression in G2M Hsiao et al. (2020). (D) Real-time qPCR validation of *Foxm1* gene expression. See also Figure S4.

At 72 h post-nephrectomy, there was significant differential expression of genes with roles in cell cycle control (Figure 1F). These included genes encoding the cyclins (e.g., *Ccna2*, *Ccnb1*, and *Ccnb2*), cyclin dependent kinases (e.g., *Cdk1*), other regulators of cell division (e.g., *Cdc20*, *Ckap2*, *Ckap2l*, *Anln*, *Ttr*, *Kif20a*, *Ube2c*, *Cenpf*, and *Top2a*) or genes known to vary with cell cycle (*Prc1* and *Tk1*). The significant differential expression of a subset of these genes (*Ube2c*, *Pclaf*, and *Nuf2*) was confirmed in qPCR assays (see Figure S4). These results support changes in cell cycle during the early compensatory hypertrophy.^8^^,^^10^^,^^25^ To examine further for alterations in genes linked to cell cycle, the count of genes with characteristic expression during the different cell cycle phases was plotted (Figure 4A). This showed striking changes with increases in the expression of genes linked to G2 and M phases at 48 and 72 h and reductions in the levels of genes linked to G1/S and M/G1 phases at 72 h. This suggests significant cell cycling during early responses to nephrectomy most in keeping with cell cycle entry of quiescent G0 cells and subsequent arrest at G2/M. Gene ontology analysis at 72 h (Figure 4B) showed marked enrichment of genes with roles in the mitotic spindle, the kinetochore, and chromosomal condensation in keeping with enhancement of cells in prophase and prometaphase stages of mitosis.^8^^,^^10^

To examine for the operation of upstream transcription factors in this alteration of expression of genes with roles in cell cycle control, a network analysis was performed using iRegulon (Figure 4C).^26^ This suggested a central role for FOXM1 in mediating such an effect and an upstream role for E2F4. Interestingly, *Foxm1* itself shows induction of mRNA expression and this regulation was confirmed with qPCR (Figure 4D).

### Single-nuclei transcriptomes of remaining kidney following unilateral nephrectomy

**Figure 5:**
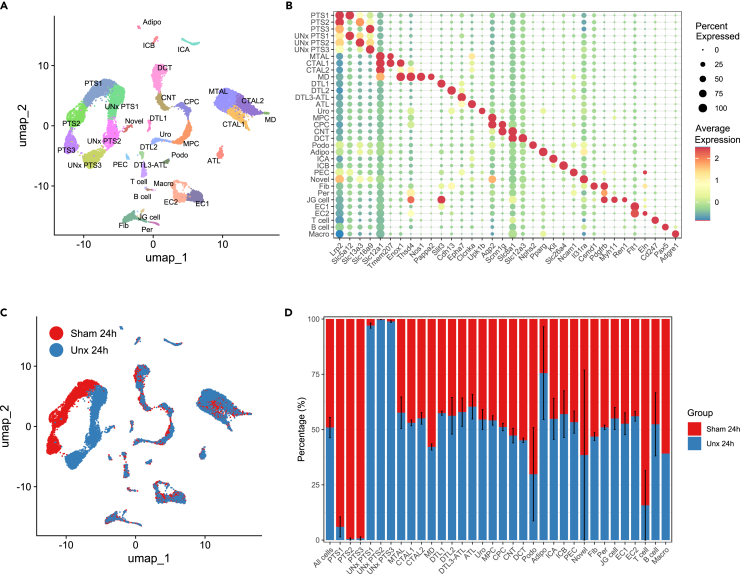
Single nucleus RNA-seq of mouse kidney undergoing CRH Mice underwent a Sham or Left uninephrectomy (Unx) surgery and remaining right kidney was collected at 24 h. A central cross-section of the kidney was used to isolate nuclei for snRNAseq (*n* = 2 mice per group at 24 h). (A) UMAP shows 31 cell types identified including Podo, podocytes; PTS, Segment of proximal tubule; PEC, parietal epithelial cells; Per, pericytes; MTAL, thick ascending limb of loop of Henle in medulla; CTAL, thick ascending limb of loop of Henle in cortex; CNT, connecting tubule; DCT, distal convoluted tubule; DTL, descending limb of loop of Henle; ATL, thin ascending limb of loop of Henle; EC, endothelial cells; Fib, fibroblasts; ICA, type A intercalated cells of collecting duct; ICB, type B intercalated cells of collecting duct; MD, macula densa; Macro, macrophages; CPC, principle cells of collecting duct in cortex; MPC, principle cells of collecting duct in medulla; Uro, urothelium; Adipo, adipocytes. (B) Shows cell types and markers used for identification of clusters. (C) UMAP showing differences in cell type grouping between Unx (blue) and Sham (red). (D) Proportion of cell types in sham and Unx. See also Figure S5.

The kidney is a complex organ with heterogeneous cells conducting specified roles. While proximal tubular cell hypertrophy is well characterized as a significant component of CRH, effects on other cell types are less understood. To better define gene expression changes in bulk kidney RNA-seq, single nucleus RNA-sequencing (snRNA-seq) was performed to investigate cell type specific gene expression changes in the remaining kidney following nephrectomy and to understand the trigger responses that facilitate the large magnitude of gene expression changes at 48 h we chose to focus on the earlier 24 h time point (Figure 5). The snRNA-seq yielded 16,500 sequenced nuclei (*n* = 2 from each group), which was completed in 2 batches. The Harmony R package was employed to correct PCA embeddings for batch effects. (See PCA plot in Figure S5A). By employing joint unbiased clustering and cell type identification using known gene markers for major kidney cell types, 33 distinct clusters were identified (Figures 5A and 5B), which was comparable between the two batches (See Figure S5B). This included the identification of differing segments of the proximal tubular cells (PTS1, PTS2, and PTS3) and importantly, rarer cell types such as those from macula densa and juxtaglomerular apparatus (JGA). The cell clusters showed similar representation and clustering when obtained from mice that had undergone uninephrectomy compared to sham operation. However, the proximal tubule clusters distinctively shifted in the UMAP analysis (Figure 5C) following nephrectomy, which is reflected in the graph showing the proportion of DEGs for each cell type (Figure 5D). Interestingly when comparing the DEGs detected on the 10× genomics platform verses bulk RNA-sequencing we saw a range of 5–18% percentage of gene overlap (Figure S5C).

### Cell specific changes occur early in CRH and are dominated by PTS and MTAL

**Figure 6:**
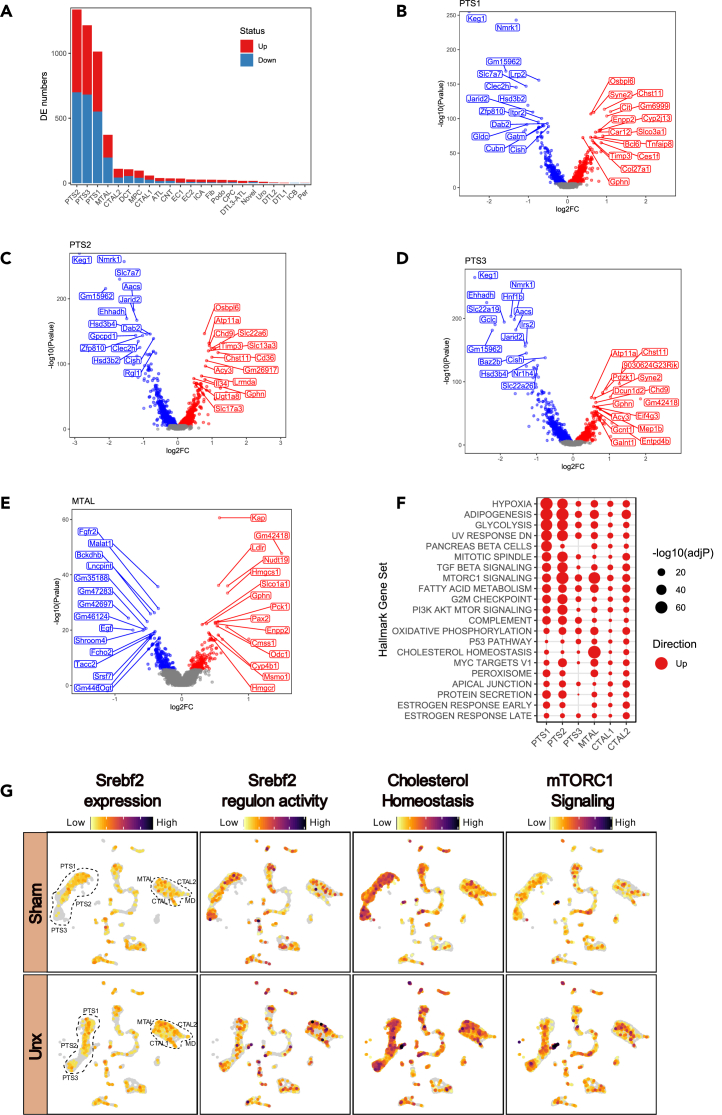
SnRNA-seq differential gene expression and hallmark analysis (A) Bar plots showing the numbers of differentially expressed genes with upregulated genes in red and downregulated gene in blue when comparing Unx to Sham (non-parametric Wilcoxon rank-sum test). Volcano plots displaying significant (adjusted *p* value <0.05) differentially expressed genes between Unx (nephrectomy) and Sham surgery at 24 h post-surgery for each cluster (B) proximal tubule segment 1 (PTS1), (C) proximal tubule segment 2 (PTS2), (D) proximal tubule segment 3 and (E) thick ascending limb of loop of Henle in medulla (MTAL). The y axis corresponds to the –log_10_(*p*-value), the x axis displays the average log_2_ fold change value. The red dots represent the upregulated genes and the blue dots represent genes whose expression is downregulated in Unx. The top 15 upregulated and top 15 downregulated genes are labeled. (F) Hallmark gene set scoring analysis was performed using the escape R package. Significant gene set was performed using Wilcoxon rank-sum test between Sham and Unx and the top 10 of each cell types were plotted. Dot plot of proximal tubules and thick ascending loop of Henle displaying significance in -log10 adjusted *p* value and red indicate the positively enriched and blue indicate negatively enriched of the gene set. (G) UMAP showing the gene expression profile of Srebf2 and its regulon activity (SCENIC) alongside signature for hallmark gene set (MSigDB) of cholesterol homeostasis and mTORC1 signaling, scored using UCell, in the kidneys of Unx and sham-operated mice collected at 24 h. PTS, segment of proximal tubule; MTAL, thick ascending limb of loop of Henle in medulla; CTAL, thick ascending limb of loop of Henle in cortex; MD, macula densa. See also Figures S6 and S7.

The most significant changes in cell specific differential gene expression occurred within the proximal tubule segments (PTS), followed by the medullary thick ascending limb (MTAL) cells (Figure 6A). Other kidney cell types displayed much fewer changes in gene expression at the 24 h time point during CRH. The significantly DEGs in PTS and MTAL clusters are shown in volcano plots (Figures 6B–6E). The top 15 regulated genes are labeled, with a third of upregulated genes in the MTAL involved in cholesterol homeostasis. This was reflected in the hallmark gene set enrichment analysis (Figure 6F), which revealed MTAL as being the unique cell type characterized by the hallmark of cholesterol homeostasis. Pathways for glycolysis, hypoxia, and adipogenesis were significantly increased in the PTS clusters from nephrectomized mice but was most prominent in segments 1 and 2. All PTS clusters showed significant increases in mTORC1 signaling, but PTS1 and PTS2 uniquely showed an increase in PI3K AKT MTOR signaling. The MTAL showed concomitant increases in mTORC1 signaling and cholesterol biosynthesis pathway. This observation that gene expression changes underlying upregulation of cholesterol biosynthesis in the bulk RNA analysis is dominated by alterations in the MTAL cells is intriguing. Although less significant gene changes involved in cholesterol biosynthesis were apparent in the PTS cells, there was an enrichment of the related signatures of adipogenesis, fatty acid metabolism, and peroxisome. Twenty-four hours following nephrectomy there were only minor changes in podocytes in regard to both gene expression and hallmark GSEA (See Figure S7).

### Cholesterol gene changes in MTAL likely driven by SREBP2

To further investigate the potential driver of cholesterol biosynthesis gene changes following nephrectomy, the transcription factor and target genes of sterol-binding protein 2 (Srebp2) were examined, as the best characterized transcriptional regulator of cholesterol biosynthesis.^27^ The mouse ortholog *Srebf2* gene expression, regulon activity, cholesterol homeostasis, and mTORC1 signaling hallmark genes were interrogated and broadcast across all cell types (Figure 6G). There were significant increases in *Srebf2* expression in the MTAL following nephrectomy (*p value* = 1.9 × 10^−9^). Less marked changes were observed in PTS cells, although PTS3 showed increased expression in a fraction of its cluster. *Srebf2* regulon activity was also most evident in the MTAL. Although cholesterol homeostasis genes appear to increase in both PTS and MTAL, the most striking difference was seen in the MTAL, while mTORC1 signaling gene expression increased across most cell clusters.

A recent report suggests that PPAR-α plays a role in compensatory renal hypertrophy,^28^ which can play roles in cholesterol metabolism and might contribute or share similarities with SREBP2 activation given overlap in their transcriptional targets and co-ordinate induction in some settings. Therefore, the expression of *Ppar-α* and of genes targeted by PPAR*-α* in our snRNA-seq data were also examined (Figure S6). *Ppar-α* and the genes it regulates were highly expressed in proximal tubules, especially in segments 1 and 2. However there were no significant changes in *Ppar-α* or its regulon when using the SCENIC analytic tool.

### SREBP2 required for IGF-1 stimulated increase in human renal tubular cell size

**Figure 7:**
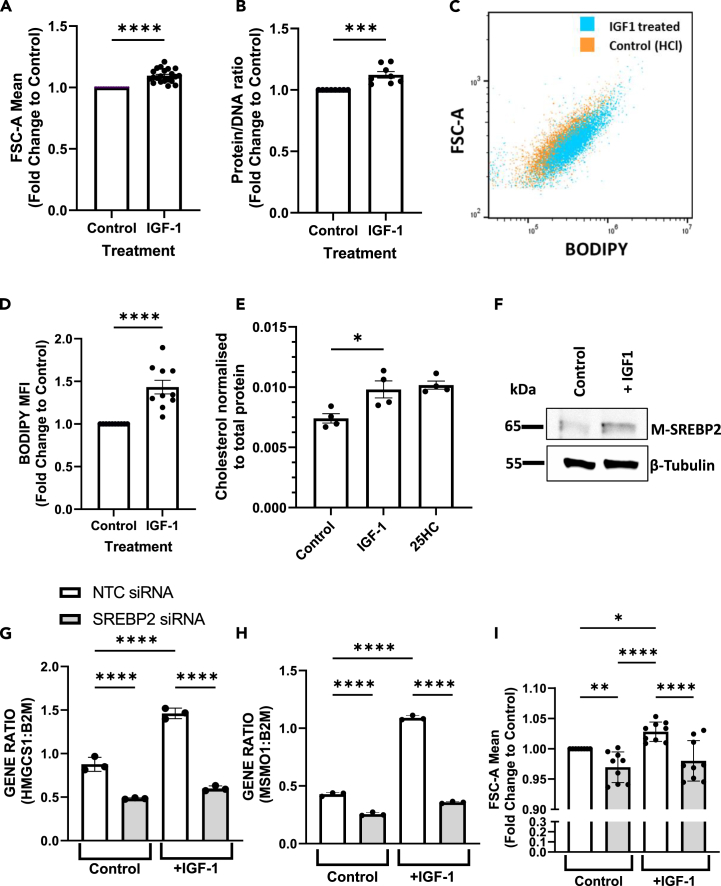
IGF-1 treatment of human proximal tubule cells concurrently increases cell size and cellular lipid content HK-2 (human proximal tubule cell line) cells were seeded and incubated for 3 days in complete media before serum starving for 48 h (serum-free DMEM/F12) and incubated for a further 48 h in the presence or absence of IGF-1 (200 ng/ml). Cells were collected and examined by Cytoflex and ImageStream flow cytometry. Cells gated on singlets were quantified for (A) cell size based on FSC-A mean, where fold change over control is plotted (*n* = 21), and (B) protein/DNA ratio (*n* = 8) of IGF-1 treated cells, expressed as fold change over control, +/− SEM error bars, one way ANOVA, ∗∗∗*p* < 0.0001, ∗∗∗∗*p* < 0.00001. (C) The flow scatterplot shows a shift in cell size (FSC-A) on the y axis and neutral lipids (BODIPY) from HK-2 cells treated with IGF-1 (blue) compared to HCl control (orange). (D) cellular neutral lipid BODIPY MFI (*n* = 10), +/− SEM error bars, one way ANOVA, ∗∗∗*p* < 0.001. (E) Intracellular cholesterol content normalized to total protein (*n* = 4), +/− SEM error bars, unpaired t-test ∗*p* < 0.05. (F) Serum starved HK2 cells were treated with IGF-1 (200 ng/ml) for 1 h. Whole cell lysates were subjected to SDS-PAGE and then transferred to nitrocellulose membranes. Membranes were probed for SREBP2, precursor protein observed at 125 kDa (P-SREBP2), the activated mature form was observed at 65 kDa (M-SREBP2). β-tubulin (55 kDa) was used as a loading control. The shown results are representative of 3 experiments. In separate experiments HK-2 cells were transfected with siRNA targeting SREBP2 or NTC control in SFM (serum-free DMEM/F12) for 24 h. Then, SFM was replaced, and treated for a further 24 h in the presence or absence of IGF-1 (200 ng/mL). Quantitative PCR was used to determine (G) *HMGCS1* gene expression and (H) *MSMO1* gene expression. Quantitative PCR results expressed as a normalized ratio to Β2M and experiment shown is representative of 2 experiments. NTC siRNA shown in white bars, SREBP2 siRNA shown gray bars. The error bars represent SD, one way ANOVA, ∗∗∗∗*p* < 0.0001. (I) Cells were collected and examined by flow cytometry, singlets were quantified for cell size based on FSC-A mean, bar graph shows fold change over control (*n* = 9). The error bars represent SEM, one way ANOVA, ∗*p* < 0.05, ∗∗*p* < 0.01,∗∗∗∗*p* < 0.0001. See also Figures S8 and S9.

Our single-cell data revealed a distinct upregulation of PI3K-AKT-MTORC signaling hallmark genes in proximal tubules. IGF-1 is recognized as a potent activator of the PI3K-AKT-MTORC signaling cascade and multiple lines of evidence suggest that IGF-1 plays a crucial role in driving compensatory renal hypertrophy. Furthermore, separate studies have demonstrated that IGF-1 signaling induces the activation of mature SREBP2 via PI3K-AKT pathway.^20^ Hence, we hypothesized that treating kidney cells with IGF-1 would lead to the activation of SREBP2, consequently resulting in kidney cell hypertrophy. To test this hypothesis, we utilized the human proximal tubule cell line HK-2 and treated them *in vitro* with IGF-1 (Figure 7). We chose this human cell line due to its translational relevance and its propensity for hypertrophic changes *in vitro*. Cells were cultured in complete media followed by 48 h serum starvation. Cells were then exposed to IGF-1 or control for 48 h and examined by flow cytometry. IGF-1 exposure led to a significant increase in cell diameter (10%) as assessed by side scatter (Figure 7A), which was accompanied by increases in protein: DNA content (Figure 7B), in keeping with hypertrophic growth. There were no changes in cell number or viability (Figures S8A and S8B). BODIPY staining was used to assess neutral lipid content and ImageStream flow cytometric analysis showed IGF-1 treatment increased BODIPY MFI (Figures 7C and 7D) in parallel with the increase in cell size. Cholesterol levels were also directly measured and showed a significant increase with IGF-1 treatment (Figure 7E). As per previous observations,^20^ IGF-1 increased SREBP2 cleavage to generate a mature form of SREBP2 (Figure 7F), suggesting that IGF-1 stimulation of proximal tubules promotes cholesterol biosynthesis during cellular hypertrophy via SREBP2 activation. In separate experiments, IGF-1 stimulation increased AKT phosphorylation (Figure S8C). To further examine the role of SREBP2 in cell size, cells were treated with siRNA targeting SREBP2 prior to IGF-1 stimulation or a control. Three siRNA targeting SREBP2 were initially tested and all three showed similar targeted knockdown of SREBP2 as shown by qPCR in Figure S8D. SiRNA1 was then selected and used for further validation SREBP2 knockdown at the protein level. This siRNA markedly reduced SREBP2 protein expression (see Figure S8E) and led to significant reduction in the expression of mRNAs encoding SREBP2 dependent genes *HMGCS1* (Figure 7G) and *MSMO1* (Figure 7H). Furthermore, SREBP2 siRNA ameliorated the IGF-1 stimulated cell size increase in HK-2 cells (Figure 7I), which was mirrored by changes in protein to DNA ratios (Figure S8F).

## Discussion

This murine compensatory renal hypertrophy model recapitulates the rapid and significant kidney growth described in other experiments and in patients undergoing nephrectomy. There are gender differences in the amplitude of compensatory growth between males and females, with male gender being a positive predictor for CRH and important contributions from estrogen signaling.^7^^,^^29^ Therefore, in this study to reduce variability due to cyclical hormonal variation in mice, the need to determine other factors contributing to CRH that are estrogen independent and to maximize the compensatory growth we chose to study male mice. Indeed they manifest substantial growth of the remaining kidney with a 27% increase in kidney: body weight ratio 2 weeks after a nephrectomy. The deliberate selection of early time points (1–3 days) has enabled a comprehensive assessment of the early transcriptional changes in the compensatory process. As observed by others, we saw evidence of mTORC activation during this early period with increased rpS6 phosphorylation and a transcriptional signature of mTORC1 activation.^9^^,^^30^

Other significant changes include increases in gene expression of glycolysis and oxidative phosphorylation, likely due to increased energy provision to support cell hypertrophy and enhanced kidney activity. At 48 h after nephrectomy, the co-ordinated increases in genes encoded by the mitochondrial genome and by some nuclear encoded genes with mitochondrial functions points to enhancement in aerobic capacity. Indeed, others have also described increases in mitochondrial metabolism, size, and number in kidneys undergoing CRH.^31^^,^^32^^,^^33^ It is possible that increased sodium reabsorption by the single kidney drives hypoxia or other signals of metabolic insufficiency that promote mitochondrial transcription and capacity.

At 24 h, there was a striking, broad induction of genes with roles in cholesterol biosynthesis and uptake and repression of cholesterol efflux genes. Sterol regulatory element-binding factor 2 (SREBP2), which co-ordinates cholesterol biosynthesis appears to be the upstream regulator further supported by snRNA-seq analysis. This may be mediated by mTORC1, which can induce *de novo* lipogenesis to fuel cell proliferation and tissue growth via AKT.^21^ However, other potential mechanisms for induction of SREBP2 in CRH include the action of ammonia^34^ or angiotensin.^35^

While it is tempting to suggest that enhanced cholesterol biosynthesis is required for the hypertrophic increases of mitochondria and membranes, other signaling roles are possible. SREBP control of cholesterol and mevalonate derivatives is essential for cell cycle progression and is coordinated with the cell cycle.^36^ IGF-1 stimulated, SREBP2 dependent enhancement of cholesterol biosynthesis was also seen in cultured renal tubular cells. While this model of IGF stimulated renal cell hypertrophy might not reflect all the *in vivo* signaling pathways underlying CRH, it does indicate that IGF-1 can enhance lipid content in hypertrophying renal tubular cells. Importantly, IGF signaling through IGF1R is dependent upon cholesterol availability indicating potential feedback for limitation of growth promotion.^37^

The changes in expression of genes with roles in cell cycle control most apparent 72 h after nephrectomy are consistent with proposals that renal cell hypertrophy is mediated in part by tubular cells transitioning from quiescence and becoming arrested in a growth phase, leading to increases in cell size and protein synthesis.^8^ This is supported by the significant gene expression signature of G2M arrest at 72 h. Upstream regulatory analysis suggested FOXM1 as a potential driver of such cell cycle alterations and is itself induced at the mRNA level. Matsushita et al. undertook cDNA microarray studies of gene expression in rat kidneys undergoing compensatory growth and suggested a role for FOXM1 in promoting hyperplasia and cell cycle progression.^38^ Furthermore, FOXM1 drives proximal tubule proliferation following AKI (acute kidney injury)^39^ and promotes compensatory liver regeneration.^40^ While growth factors such as IGFs might drive some cells to exit quiescence, via FOXM1, and enter the cell cycle, the signals that lead to subsequent cell-cycle arrest are less clear. The upstream analysis also implicated E2F4 in the control of cell cycle at 72 h and E2F4 can promote cell-cycle arrest, although its effects are complex and context dependent.^41^ There is also intriguing crosstalk between cholesterol metabolism and cell cycle control which may connect the early changes seen in the expression of cholesterol biosynthetic enzymes with cell cycle regulation.^37^ These and the other alterations in cellular metabolism suggested by these data may be required to achieve exit from quiescence.^42^^,^^43^

The many heterogeneous cell types in the kidney make it difficult to delineate signals in bulk RNA-seq and single cell studies are providing fresh insights into kidney cell heterogeneity, physiology, and pathophysiology.^44^^,^^45^^,^^46^^,^^47^ In this study single nuclei RNA-seq was utilized to minimize tissue dissociation bias, to maximize the capture of kidney cell types for sequencing and understand the cellular localization of the pathway changes at 24 h. The success of this approach is indicated by the 33 different cell types that were identified including rarer cell types such as macula densa. Proximal tubules showed the highest differential gene expression changes, followed by MTAL. Despite modest overlap of DEGs between snRNA-seq and bulk RNA-seq, likely due to platform technical differences^48^ and 10X-based data gene dropout issue,^49^ snRNA-seq identified MTAL as the cells responsible for the cholesterol biosynthesis gene signature seen in the bulk data. The role of cholesterol in MTAL function is not fully characterized, though functional effects of dietary cholesterol limitation and enrichment on tubular function have been described and the role of lipogenesis and fatty acid oxidation in kidney disease has recently been recognized.^45^^,^^50^^,^^51^^,^^52^

During the course of this work, Kikuchi and colleagues utilized models of mouse nephrectomy and undertook ATAC-seq, RNA-seq and proteomic studies of micro-dissected proximal tubules from hypertrophying kidneys. They also saw activation of mTORC1 signaling, oxidative phosphorylation, changes in fatty acid metabolism, and cholesterol homeostasis.^28^ Upstream analysis suggested activation by SREBF2 and E2F, but a more prominent role for PPARα and their ATAC-seq studies suggest a direct transcriptional effect in proximal tubules at least. Our snRNA-seq data were therefore interrogated for PPARα and target gene expression. However, there was no or minimal induction of PPARα and its target genes in the proximal tubular or MTAL cells and was much less prominent than activation by SREBF2. An interrelationship between PPAR-α and SREBPs has been proposed,^53^ where activation of PPAR-α, can lower cholesterol synthesis and concentration via SREBP2.^54^ Other models have shown PPAR-α activation can upregulate cholesterol biosynthesis genes,^55^ demonstrating that this interrelationship is complex and we agree with the comments of Kikuchi and colleagues that growing evidence points to the role of PPARα in regulation of lipogenesis that is dependent on the SREBP family. The signatures of mTORC1 signaling, oxidative phosphorylation, and cholesterol homeostasis are shared with the results in this study as are GSEA enrichment of mitotic spindle, G2M checkpoint and E2F targets despite the differing methodologies. They also provided additional evidence from genetic, proteomic, lipidomic, and ATAC-seq experiments for the importance of changes in lipid metabolism and PPARα action in compensatory renal growth. It will be important to understand the cell and time specific alterations of these transcription factors and their upstream mechanism of activation in compensatory growth and how enhancement of specific lipid pathways are beneficial to kidney function in some circumstances but deleterious in others.^56^^,^^57^

Studies to further explore these observations could be undertaken in differing animal species, in both genders and in animals with genetic and pharmacological manipulations of cholesterol biosynthesis and its upstream regulators including SREBP2. A potential translational application of this work is in the use of specific nutritional perioperative interventions that could enhance cholesterol biosynthesis, mitochondrial activity, and mTOR activity in patients undergoing nephrectomy for cancer or those donating or receiving transplant kidneys.

In conclusion, these observations are consistent with a vital role for IGF signaling, cell cycle changes and enhanced cholesterol biosynthesis during CRH, but will require further exploration of their relevance to compensatory hypertrophy in human kidneys and the extent to which they have an obligatory role in kidney growth. This study also highlights the importance of using single cell transcriptomics to decipher the co-ordinated changes that occur in the remaining kidney following a nephrectomy.

### Limitations of the study

These studies exploring the underlying mRNA changes in kidneys early in the compensatory growth response have been restricted to one species and broader applicability to other animals and responses in humans is required. While independent validation has been obtained for many of these transcript changes and separate single cell studies undertaken, the extent to which such changes result in alterations in protein abundance is less clear. For some of the pathways perturbed in these growth responses bioinformatic predictions have been compelling but will require further experimental validation. Defining initiating mechanisms and very proximal signaling and distinguishing specific growth responses from the effects of surgical interventions remains challenging.

## STAR★Methods

### Key resources table

**Table:** 

REAGENT or RESOURCE	SOURCE	IDENTIFIER
**Antibodies**		

SREBP2 antibody (Clone IC6, BD)	BD Pharmingen	Cat#557037; RRID: AB_396560
Anti-Phospho-S6 Ribosomal Protein (Ser240/244; Clone D68F8)	Cell signaling	Cat#5364S; RRID:AB_10694233
Anti-S6 Ribosomal Protein (Clone:5G10),	Cell signaling	Cat# 2217S; RRID:AB_331355
Anti-pAkt (Ser473: Clone: 587F11),	Cell signaling	Cat#4051S; RRID:AB_331158
Anti- total AKT	Cell signaling	Cat#9272S; RRID:AB_329827
β-Tubulin (Clone: 9F3)	Cell signaling	Cat#2128S; RRID:AB_823664
β-Actin (Clone: 8H10D10)	Cell signaling	Cat#3700S; RRID:AB_2242334

**Chemicals, peptides, and recombinant proteins**		

Recombinant Human insulin like growth-1 (IGF-1) Receptor Grade	GroPep Bioreagents	Cat#CU100
BODIPY (10 ng/mL)	Invitrogen	D3922

**Critical commercial assays**		

Illumina TruSeq Stranded mRNA library preparation kit	Illumina	20020594
Chromium Single Cell 3′ Reagent Kit v3	10x Genomics	Cat#1000269
Cholesterol assay kit	Abcam	Cat#ab65390

**Deposited data**		

ProteomeXchange Consortium PRIDE partner repository	This Paper	PXD047516
Data for bulk RNA-Seq and snRNA-Seq	This Paper	GSE241213

**Experimental models: cell lines**		

Human: Human Kidney 2 (HK2)	ATCC	CRL-2190

**Experimental models: organisms/strains**		

Mouse: C57BL/6J	College of Medicine and Public Health Flinders University Animal Facility	RRID:IMSR_JAX:000664

**Oligonucleotides**		

Abca1 (Mm00442646_m1)	Life Technologies	Cat# 4331182
B2M (Mm00437762_m1)	Life Technologies	Cat# 4331182
Foxm1 (Mm00514924_m1)	Life Technologies	Cat# 4331182
Hmgcs2 (Mm00550050_m1)	Life Technologies	Cat# 4331182
Lipg (Mm00495368_m1)	Life Technologies	Cat# 4331182
Pcsk9 (Mm01263610_m1)	Life Technologies	Cat# 4331182
siRNA targeting: SREBP2	IDT	Cat#hs.Ri.SREBF2.13.1
Primer: SREBP2 forward: TGTGTATGTCCTGTGCCTTTTC	*Kondo* et al.^58^	N/A
Primer: SREBP2 Reverse: TGGGACACAGTGACTGATTGAT	*Kondo* et al.^58^	N/A
See Table S7 for additional primers	N/A	N/A

**Software and algorithms**		

FastQC (v0.11.8)	http://www.bioinformatics.babraham.ac.uk/projects/fastqc	N/A
AdapterRemoval version 2.3.2	Schubert et al.^59^	N/A
Salmon version 1.5.0	Patro et al.^60^	N/A
R statistical software environment version 4.10	R Core Team et al.^61^	N/A
EdgeR	Robinson et al.^62^	N/A
DIA Data Analysis: Spectronaut™ (version 14.11.210528.47784)	Biognosis	N/A

### Resource availability

#### Lead contact

Further information and requests for resources and reagents should be directed to and will be fulfilled by the lead contact, Jonathan Gleadle (Jonathan.gleadle@flinders.edu.au).

#### Materials availability

This study did not generate any new or unique reagents.

#### Data and code availability

• The data supporting the findings for bulk RNA-Seq and snRNA-Seq data are openly available at https://www.ncbi.nlm.nih.gov/geo/query/acc.cgi?acc=GSE241213 (GEO: GSE214213). Mass spectrometry proteomics data were deposited to ProteomeXchange Consortium via PRIDE, ProteomeXchange accession: PXD047516 and is publically available at http://www.ebi.ac.uk/pride/archive/projects/PXD047516.
• No original code was generated for this study. All code used for analysis was properly cited in the STAR methods.
• Any additional information required to reanalyze the data reported in this paper is available from the lead contact upon request.

### Experimental model and study participant details

#### Mice

Male C57/BL6J mice of 8–12 weeks of age (bred and supplied by Flinders University Animal Facility, Bedford Park, SA, Australia) were randomized and housed in 2–5 per cage and were exposed to 12-h light/dark cycles in temperature-controlled environment and had *ad libitum* access to food (23% protein, 6% fat, 5% fiber, Rat and Mouse Premium Breeder Diet, Gordon’s Specialty Stockfeeds, Yanderra, NSW, Australia) and water.

#### Human cell line

The Human Kidney 2 (HK2) cell line (CRL-2190) was obtained from American Type Culture Collection (ATCC, Manassas, USA) is a proximal tubular cell line derived from normal kidney of male gender. Cultures were maintained in DMEM/F12 media (Invitrogen) supplemented with 10% Fetal Calf Serum (FCS) at 37°C under 5% CO_2._

#### Study approval

All animal procedures and protocols were approved by the Flinders University College of Medicine and Public Health Animal Welfare Committee (Approval number: 955/18) and were conducted in accordance with the Australian code for care and use of animals for scientific purposes (NHMRC).

### Method details

#### Animal surgery

Mice were anesthetized using 1.5–2.5% isoflurane/oxygen (1.5 L/min), the left kidney was accessed with a mid-lateral skin incision and exposed. Adherent fat and the adrenal gland were bluntly dissected from the cranial pole. Renal blood supply was clamped and doubly ligated (Prolene 4-0). The clamp was removed after kidney removal, and the abdominal wall was closed by simple interrupted stitches (Prolene 4-0). Skin was closed with wound clips. Mice were given subcutaneous 0.5mL 0.9% sterile saline and Buprenorphine (0.05 mg/kg) at time of surgery. Pain relief was given every 8–12 h post-surgery as required for the first 24 h. Sham operations were performed similarly without kidney removal.

#### Bulk RNA sequencing (RNA-Seq)

At 24, 48 and 72 h post nephrectomy, the remaining right kidney was collected, and RNA was extracted and quantified from mid-cross sectional kidney samples. Library preparation was performed using the Illumina TruSeq Stranded mRNA library preparation kit and sequenced using the NovaSeq system (Illumina) platform with 150 bp paired end read length.

#### Quality control assessment

RNA-seq raw data in FASTQ format from individual libraries were uploaded to Deep Thought High Performance Compute Server, hosted by Flinders University for processing. FastQC (v0.11.8) was used for checking the quality of the FASTQ file (Available at: http://www.bioinformatics.babraham.ac.uk/projects/fastqc). After inspection of data quality, low quality bases and Illumina Truseq adapters were then removed using AdapterRemoval version 2.3.2^59^ with parameters --trimns --minquality 30 --minlength 50.

#### Pseudoalignment

Quantification of transcript expression in RNA-seq data were conducted using Salmon version 1.5.0 using parameters -libType A --seqBias --numBootstraps 50 –validate Mappings.^60^ First, a decoy-aware transcriptome index was built using transcript sequences and primary assembly genome sequences from GENCODE Mouse Release M27.^63^ RNA-seq reads were then pseudoaligned to the transcriptome index and output was generated in a sub-directory containing a simple TSV format file listing the name of each transcript, its length, effective length, and its abundance in terms of Transcripts Per Million (TPM) as well as estimated number of reads originating from each transcript. After the quantification, results were imported into R statistical software environment version 4.10 (R Core Team, 2020)^61^ for downstream analysis.

#### Differential gene expression analysis

Differential expression (DE) analysis was carried out using edgeR.^62^ Transcript counts from Salmon output for a series of samples were imported into R and the bootstrap samples were used to estimate the mapping uncertainty for each transcript. Transcripts were then associated with gene IDs for gene-level summarization. Lowly expressed genes were filtered to retain genes that expressed at least 1 count per million in the smallest group of samples. Gene expression distribution was normalized by the method of trimmed mean of M-values (TMM)^62^ using the calcNormFactors function in edgeR. Principal component analysis (PCA) plot was used to inspect if samples belonged to the same group. Where sample-level variation was evident from inspections of the PCA plot, the voomWithQualityWeights^64^ function was used to simultaneously incorporate sample-level weights together with the abundance dependent weights estimated by voom transformation.^65^ A linear model was then fitted for each gene, using the logCPM post-voom transformation for each gene, the design containing the experimental design, and the contrasts which specify the pairwise comparisons to be tested. The treat step applies Bayesian statistics to “borrow” information across the individual moderated t-tests for each gene, increasing power to detect DEGs. False Discovery Rate (FDR) method was performed for multiple testing correction, and the significance cut-off for the adjusted *p*-value was set at 0.05. The DEGs from the limma-voom analysis were obtained using the topTreat function from limma.^66^ This gives a spreadsheet-like table where each row is a gene and the columns contain information including the *p*-value (P.Value), FDR-adjusted *p*-value (adj.P.Val), t-statistic (t), log2 fold change (logFC) and more. Genes showing significant differential expression were extracted for visualization, pathway enrichment analysis, data intersection and modular co-expression analysis.

#### Hallmark gene set enrichment analysis

To examine for patterns of gene expression at each timepoint, FRY gene set test was performed to assess whether specific Hallmark gene sets from Molecular Signatures Database (MSigDB) v7.5.1 were significantly enriched.^67^ Briefly, FRY uses Monte-Carlo system of randomization known as rotation to test whether a set of genes is differentially expressed, assessing the complete set of genes. Gene set tests consider all the genes in the specified set and do not depend on any pre-emptive significance cut-off. Selected hallmark gene sets with an adjusted *p*-value <0.01 cut-off from each group were then chosen for dot plot visualization.

#### Quantitative real-time PCR

Messenger RNA expression was assessed by quantitative real-time PCR (qPCR) using TaqMan gene expression assays (Applied Biosystems). Complementary DNA (cDNA) was synthesized from 2.5 μg total RNA using the SuperScript IV VILO Master Mix (Thermo 11756050) kit as per the manufacturer’s instructions. qPCR was performed in a Qiagen Rotor-Gene Q (Qiagen). Each qPCR was performed in triplicate and contained 2μL of a 1:5 dilution of cDNA, 1× TaqMan Fast Advanced Master Mix (Applied Biosystems 4444557) and 1× TaqMan Gene Expression Assay primer and hydrolysis probe mix (assay IDs: Mm00442646_m1 (Abca1: ATP binding cassette subfamily A member 1), Mm00437762_m1 (B2M: beta-2 microglobulin); Mm00514924_m1 (Foxm1: forkhead box M1), Mm00550050_m1 (Hmgcs2: 3-hydroxy-3-methylglutaryl-CoA synthase 2), Mm00495368_m1 (Lipg: lipase, endothelial), Mm01283863_m1 (Nuf2: NUF2 component of NDC80 kinetochore complex) Mm00783087_s1 (Pclaf: PCNA clamp associated factor), Mm01263610_m1 (Pcsk9: proprotein convertase subtilisin/kexin type 9), Mm00835439_g1 (Ube2c: ubiquitin-conjugating enzyme E2 C) Applied Biosystems). The 10μL qPCR reactions were cycled at 95°C for 20s followed by 50 cycles of 95°C 5s and 60°C 30s. The qPCR data were analyzed in the Rotor-Gene Q Series Software v2.3.4 (Qiagen) and the cycle threshold (Ct) value was used to assess gene expression levels. The qPCR data were grouped by timepoint and treatment group and is represented as standard error of the mean (SEM). Unpaired 2-tailed Student’s t test was used to compare two groups and a *p* value less than 0.05 was considered significant. For SREBP2 target gene analysis used previously published primers for HMGCS1 and MSMO1,^58^ see Table S7 for primer sequences.

#### Mass spectrometry

##### Sample preparation

1 mg (approx.) piece of kidney tissue was collected and stored at −80°C. 1 mL of 8 mM Tris pH 8.0 buffer containing a protease inhibitor cocktail (cOmplete ULTRA, Roche) was added to each of the thawed kidney samples in a dounce homogeniser and the tissue was homogenized using 20 strokes. n-Dodecyl-β-D-Maltopyranoside (Anatrace) was added to the homogenates to a final concentration of 0.1% and incubated at 37°C for 10 min before centrifuging at 150,000 g at 4°C for 40 min using an Optima MAX-TL Ultracentrifuge (Beckman Coulter). The supernatants were collected, and protein estimation was performed using an EZQ Protein Quantitation Kit (Invitrogen), following the manufacturers protocol. The assay paper was imaged using a BioRad GelDoc EZ imager with the Image Lab SYPRO Ruby protocol. Image Lab volume tools was used to estimate protein concentrations.

##### Protein digestion

Proteins were reduced with tris (2-carboxyethyl) phosphine (TCEP, 100 mM, 30 min, 56°C) and alkylated in the dark with chloroacetamide (200 mM, 30 min, RT) (Sigma-Aldrich, St Louis, USA). The pH of the reduced and alkylated protein solutions were adjusted to pH 8.0 by the addition of 1M Tris pH 8.0 and digested using trypsin (1:20 enzyme-to-substrate ratio) (Promega, Madison, USA) and incubated overnight at 37°C.

##### Liquid chromatography mass spectrometry

Peptides were analyzed with a Dionex Ultimate 3000 UPLC coupled with a Thermo, Exploris 480 tandem mass spectrometer (Thermo Fisher Scientific, Waltham, Massachusetts, USA). An inhouse analytical column created from 75 μm inner diameter fused silica capillary with an integrated pulled tip emitter, packed with 1.9 μm ReproSil-Pur C18 beads (Dr. Maisch, Ammerbuch, Germany) to 40 cm, coupled with a PepMap 100 trap cartridge (0.3 × 5 mm, 5 μm C18, Thermo Fischer) were used. Mobile phase A was 0.1% formic acid in water and mobile phase B was 0.1% formic acid in 80% acetonitrile. For each injection, 1 μg peptides were loaded and separated using a 60 min gradient from 3 to 25% mobile phase B, followed by a 40 min washing and equilibration gradient.

##### Spectral library generation

5 μg of each 24 h Unx and sham protein digests were pooled to create two pooled peptide samples (24 h Unx and 24 h Sham). Each of the digest pools were fractionated using a Pierce High pH Reversed-Phase Peptide Fractionation Kit (Thermo Scientific) following the manufacturers protocol. Each of the pooled peptide samples were bound to an equilibrated fractionation spin column that were then washed with 300 μL of water before peptides were consecutively eluted with eight elution solutions containing 5, 7.5, 10, 12.5, 15, 17.5, 20 and 50% acetonitrile in 0.1% triethylamine (TEA). The volumes of the elution fractions were reduced to 10 μL using a vacuum centrifuge, then acidified by the addition of 1 μL 10% formic acid prior to mass spectrometry. 2 μL of each fraction from the sample pools were analyzed using data dependent acquisition (DDA) utilizing a top 38 instrument method with a FAIMS unit installed. Briefly, ms1 scans were performed using an orbitrap resolution of 60,000 and FAIMS compensation voltages of −50V and −70V. A normalized AGC target of 3e6 with auto maximum injection time mode used. An intensity threshold of 5e4 and dynamic exclusion time of 20 s was employed for all data dependent ms2 scans that were acquired at 15,000 resolution, AGC target 7.5e4, 30% normalized collision energy (NCE) in the HCD cell, with auto maximum injection time mode used.

##### Quantitative shotgun data independent acquisition (DIA) mass spectrometry

For Data Independent Acquisition (DIA) runs, the Thermo Exploris 480 was configured to acquire 54 15 m/z precursor isolation windows (349.5–1150.5 m/z), with a 1 m/z window overlap. An ms2 resolution of 15,000, AGC target 1e6, auto maximum inject time, and normalized HDC collision energy of 30% was employed for all DIA scans. Precursor spectra over a 350–1200 m/z mass range were acquired prior to DIA scans with a resolution of 30,000, standard AGC target and auto maximum inject time were used for all full scan MS spectra.

##### DIA data analysis

Spectronaut (version 14.11.210528.47784, Biognosis) was used for both spectral library generation and DIA data analysis. Factory default settings were used for all analysis steps. Mass spectrometry proteomics data have been deposited to the ProteomeXchange Consortium via PRIDE partner repository ProteomeXchange accession: PXD047516.

#### Single nuclei RNA-Seq

##### Sample preparation

Right kidneys were harvested from mice 24 h post-sham operation and unilateral nephrectomy (*n* = 2 per group). A small mid cross-section of the kidney was excised and processed for nuclei isolation as per published protocol.^68^ Briefly, kidney section was minced and homogenized in EZ lysis buffer. The homogenate was filtered through a 40 μm cell strainer and centrifuged to pellet the nuclei. The nuclei pellet was resuspended and layered onto a sucrose gradient and centrifuged to separate and remove any other cellular components and debris. The purified nuclei were resuspended in an appropriate volume of resuspension buffer. Single nuclei were then sorted based on DAPI fluorescence using a flow cytometer. The sorted nuclei were counted using a CellDrop FL Automated Cell Counter (DeNovix) to determine the concentration. A total of good quality 16,500 nuclei post data filtering were sampled from kidney sections obtained from two distinct groups of mice: sham-operated and Unx mice. The sample size was divided into two batches, with each batch representing one mouse from each group. In the first batch, the Unx mouse kidney section yielded 5,151 nuclei, while the sham mouse kidney section provided 5,342 nuclei. In the second batch, the sham mouse kidney section contained 2,641 nuclei, and the Unx mouse kidney section had 3,366 nuclei. This sample size allocation allows for a comprehensive analysis of both sham and Unx groups, ensuring sufficient statistical power to detect potential differences between cell clusters. The use of two mice from each group in separate batches also helps control for potential batch-to-batch variations, enhancing the reliability of the study’s findings and conclusions. The single nuclei suspension was loaded onto a Chromium Controller (10× Genomics) for single nuclei encapsulation, barcoding, and library preparation using the Chromium Single Cell 3′ Reagent Kit v3 (10× Genomics) following the manufacturer’s instructions. The prepared libraries were sequenced on an MGI FCL sequencer.

##### Data processing and analysis

Raw sequencing data were processed using the Alevin-fry pipeline with the mouse reference genome (mm10) splice reference and index for pseudo-alignment and gene quantification was carried out using Alevin-fry’s quant mode. The resulting gene expression matrix was imported into Seurat for single-cell RNA sequencing data analysis. Seurat was used for quality control, filtering out low-quality cells and potential doublets. The filtered data were then SCTransform normalized and to regress out percentage of mitochondria and ribosomal transcript per cell. Principal component analysis (PCA) was performed on the variable genes, followed by unsupervised clustering using the Louvain algorithm. Harmony was used to remove the influence of batch effects.^69^ Uniform Manifold Approximation and Projection (UMAP) was employed for dimensionality reduction and visualization of the clusters. Differential gene expression analysis was performed on individual clusters using Seurat’s FindMarkers function to perform a Wilcoxon Rank-Sum test by default (adjusted *p*-value <0.05). The results were used to identify marker genes and characterize the cell types present in the kidney tissue. Single-cell gene set scoring analysis was performed using ssGSEA method within the ESCAPE (Enrichment of Single-Cell Annotations using Pathway Expression) package in R using the hallmark gene set.^70^ The output from ESCAPE included associated enrichment scores and *p*-values for each cell. SCENIC pipeline was employed to identify activity of transcription factors (TFs).^71^ Briefly, co-expression network of TFs and target genes were calculated to infer regulatory relationships and provided an output of regulon activity for each transcription factor.

#### Western blot

##### Chemiluminescence

Cell pellets were lysed with 1%SDS lysis buffer (10 mM Tris HCL [pH7.6], 100 mM NaCl, 1%[w/v] SDS). Protein sample was dissolved in 4× Laemmli sample buffer containing 200 mM Dithiothreitol (DTT) at 95°C for 5 min. Protein samples were then resolved by sodium dodecyl sulphate–polyacrylamide gel electrophoresis (SDS-PAGE) using 7.5% precast gradient gel (BioRad) and transferred onto Nitrocellulose membranes (BioRad) using the semi-dry transblot system (BioRad). Blots were blocked with 5% skim milk in PBS for 1 h at room temperature before probing with primary antibody overnight at 4°C. Blots were incubated with secondary antibodies conjugated to HRP for 1 h at room temperature (RT). Proteins were visualized via chemiluminescence using the Clarity reagent as per manufacturer’s instructions (BioRad) and imaged on the ChemiDoc Imaging System (BioRad, Hercules, CA, USA). SREBP2 antibody (Clone IC6, BD) was used at 1:250. B-Tubulin was used at 1:1000 (Cell signaling).

##### IR visualization

For kidney tissues were lysed in RIPA buffer (Thermo Scientific) and homogenized using a Dounce homogeniser (Sigma-Aldrich). Protein sample (10 μg) was dissolved in 2× Laemmli sample buffer containing 100 mM Dithiothreitol (DTT) at 70°C for 10 min. Protein samples were then resolved by sodium dodecyl sulphate–polyacrylamide gel electrophoresis (SDS-PAGE) using Any KdTM precast gradient gel (BioRad) and transferred onto low fluorescence Polyvinylidene Difluoride (PVDF) membranes (Thermo Scientific) using the semi-dry transblot system (BioRad). Blots were blocked with 2% BSA in PBST (0.05% Tween 20 in phosphate buffered saline (PBS) for 2 h at room temperature before probing with primary antibody overnight at 4°C. Proteins were visualized, by incubating blots with secondary antibodies conjugated to IRDye for 1 h at room temperature (RT). Blots were then scanned on the Odyssey Imaging System (Licor, Lincoln,NE, USA). The following primary antibodies were used: anti-Phospho-S6 Ribosomal Protein (Ser240/244; Clone D68F8) and anti-S6 Ribosomal Protein (Clone:5G10), anti-pAkt (Ser473: Clone: 587F11), anti-total AKT (#9272), β-Tubulin (Clone: 9F3), β-Actin (Clone: 8H10D10) antibodies were all purchased from Cell Signaling and were used at 1:1000.

#### Cell culture experiments

##### Treatment

HK2 cells were seeded at 1 × 10^4^ cells/cm^2^ and incubated for 3 days in 10% FCS DMEM/F12 (Invitrogen) (supplemented with 15 mM HEPES, 1× Pen-Strep, 1× Glutamax) and were serum-starved for 48 h in serum-free DMEM/F12 complete media. The cells were then treated with either Control solution (control, 2 μM HCl, 0.8 μg BSA) or IGF-1 (200 ng/mL from GroPep Bioreagents, 2 μM HCl, 0.8 μg BSA) in fresh serum-free media, for a further 48 h.

##### Flow cytometry staining

Live cells were collected and stained for BODIPY (10 ng/mL) for 1 h on ice before data acquisition with Cytoflex and ImageStream flow cytometry for cell size measurement (FSC-A) and neutral lipid content. For protein/DNA staining, cells were fixed for 20 min in ice-cold 4% PFA solution at 4°C. Cells were then washed in PBS and permeabilized using 0.5% Triton X- for 10 min at room temperature. After permeabilization, NHS-ester (400 ng/mL) and Hoechst (10μg/mL) were added and incubated for a further 45 min before data acquisition on Cytoflex for protein and DNA MFI.

##### Cholesterol quantification

Cellular cholesterol levels were measured in HK2 cells using a cholesterol assay kit (Abcam #ab65390) following the manufacturer’s instructions. Briefly, cells were lysed in Cholesterol Assay Buffer, and the cholesterol content was quantified by measuring the absorbance at a Ex/Em = 535/587 nm for fluorometric assay using a microplate reader. Protein concentration was measured using the Qubit assay for protein (Thermo Fisher Scientific Inc.; #Q33211), and cholesterol levels were normalized to total protein content. In separate experiments cholesterol content was quantified (See STAR methods for details).

##### SiRNA transfection

HK2 cells were seeded in 6-well plates at a density of 1.5 × 10^5^ cells per well and allowed to reach 50–70% confluence before transfection. Cells were transfected with 10 nM SREBP2-targeting siRNA (SREBP2 siRNA; IDT: hs.Ri.SREBF2.13.1) or non-targeting control siRNA (IDT) using Lipofectamine 3000 (Invitrogen). Total RNA and protein were isolated from transfected HK2 cells at 48 h post-transfection.

### Quantification and statistical analysis

For pairwise comparisons, a two-tailed t-test was utilized. Mann-Whitney non-parametric test for histograms of weighted values was also used. Significance is expressed as a two-tailed *p*-value <0.05. The number of samples for each experiment is noted in the figure legends.
