# Supplementary material for: The transcriptome of early compensatory kidney growth reveals cell and time specific responses
Source: iScience. 2024 Jul 27;27(9):110608. doi: 10.1016/j.isci.2024.110608 (PMC11363579; doi:10.1016/j.isci.2024.110608)
Supplement: Document S1. Figures S1–S9 and Tables S1–S7 [file mmc1.pdf]

iScience, Volume *27*

Supplemental information

The transcriptome of early compensatory kidney growth reveals cell and time specific responses

Darling M. Rojas-Canales, Soon Wei Wong, Elise J. Tucker, Anthony O. Fedele, Kym McNicholas, Anne-Sophie Mehdorn, and Jonathan M. Gleadle

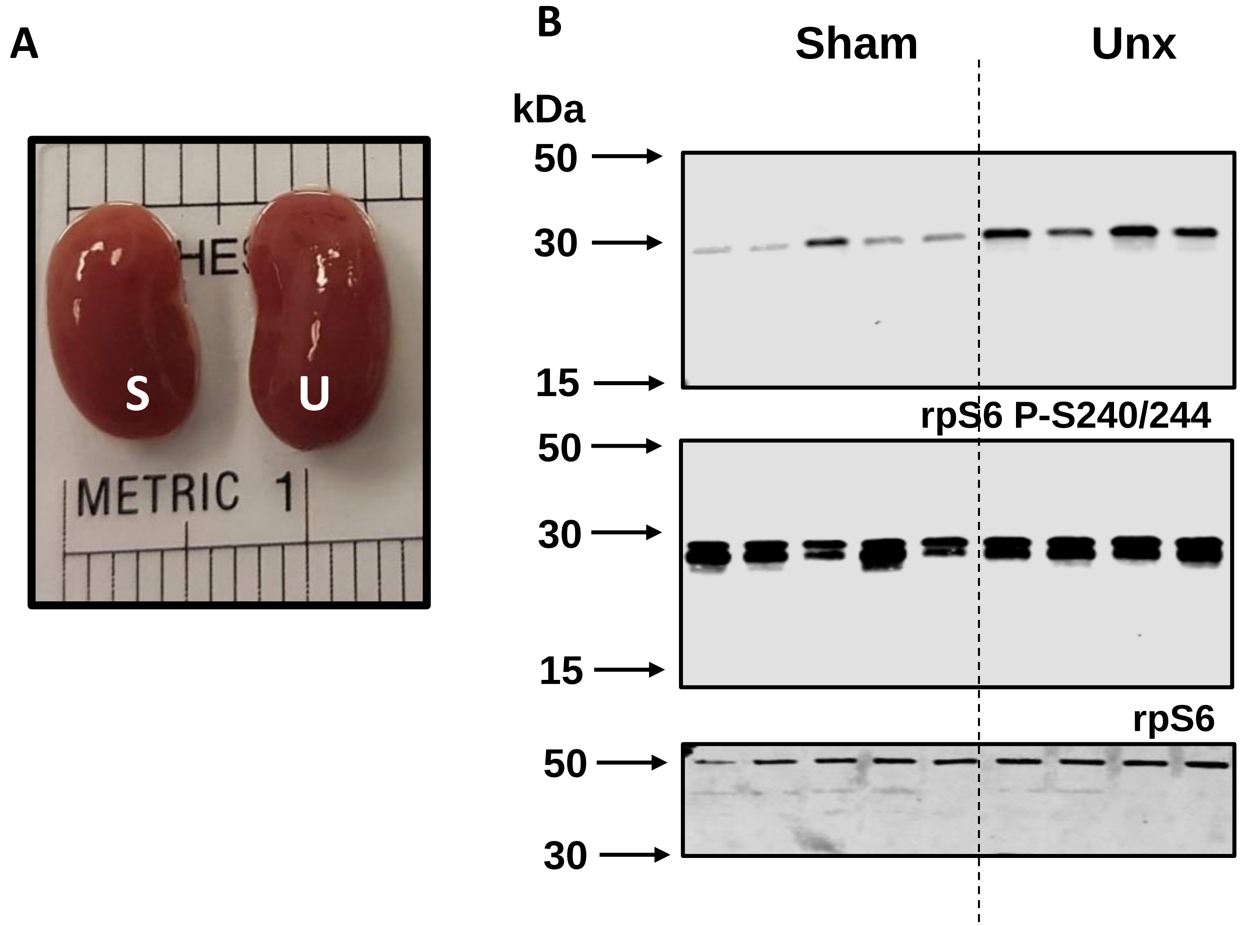

Figure S1: Protein expression analysis of rpS6 P-S240/244 and total rpS6 in mice (C57BL6) following a unilateral nephrectomy, related to Figure 1. Mice (C57BL6) were subjected to either sham surgery or left unilateral nephrectomy (Unx). **(A)** Two weeks following a left kidney nephrectomy the remanent kidney (labelled U) was photographed to demonstrate relative whole kidney size change when compared to control sham operation (labelled S). **(B)** At 24 hours post-surgery, the remaining kidney was harvested (4-5 mice per group). Whole tissue lysates were prepared and separated by SDS-PAGE. Subsequently, the proteins were transferred to membranes for immunoblotting. The membranes were probed with specific antibodies against rpS6 phosphorylated at S240/244 (rpS6 P-S240/244) and total rpS6. β-tubulin (55 kDa) was used as a loading control for normalization. The data indicates increases in rpS6 phosphorylation following unilateral nephrectomy, confirming early mTORC pathway signalling.

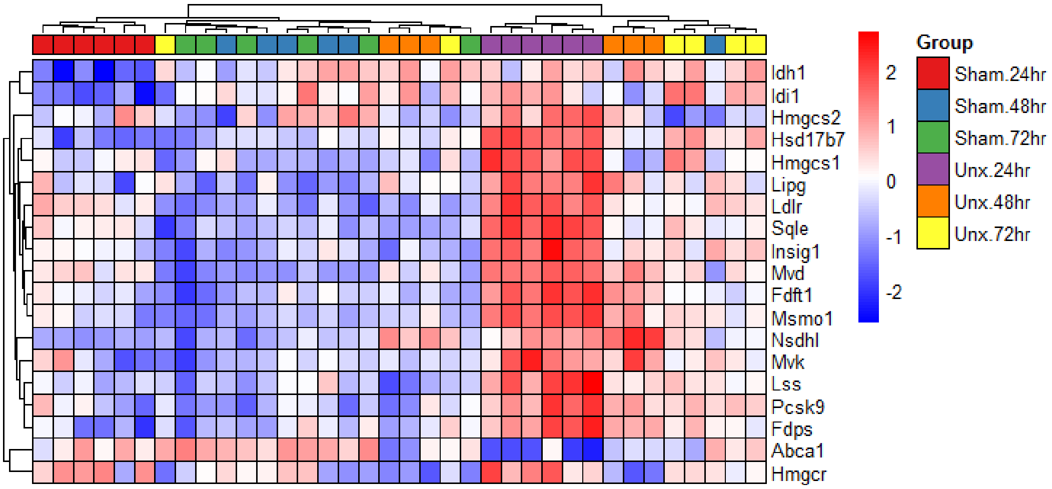

Figure S2: Heatmap of genes with roles in cholesterol biosynthesis and transport across all timepoints, related to Figure 2. Showing samples from individual mice and analysed by unsupervised clustering and grouped together by similarity in patterns of gene expression.

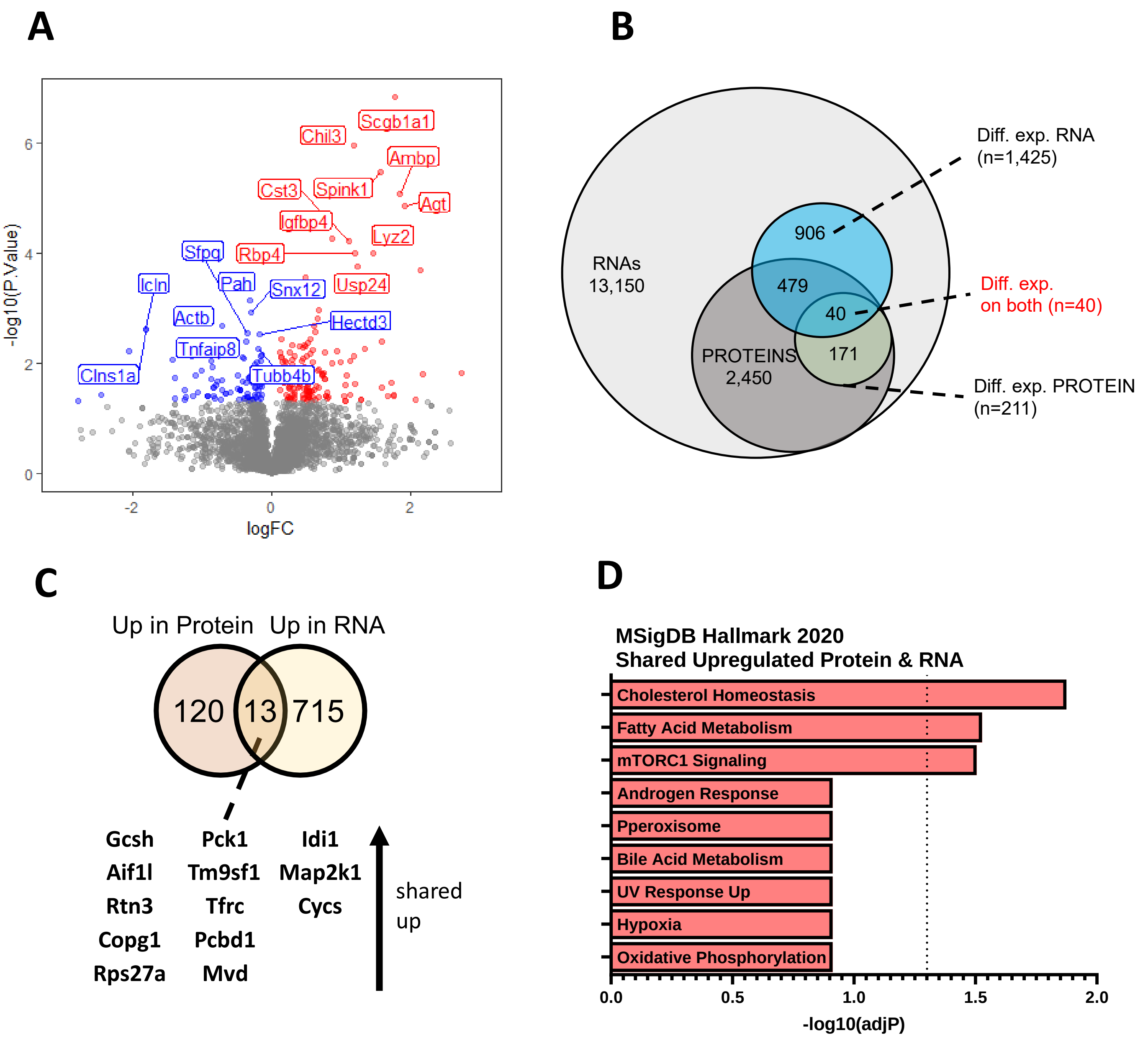

Figure S3: Untargeted Mass Spectrometric analysis of kidney tissue lysates 24 hours post surgery, related to Figure 2. Sham surgery (n=6) was compared to nephrectomy (n=6). **(A)** Volcano plot showing the top 10 upregulated and top 10 downregulated protein at 24h. **(B)** Venn diagram showing the overlap in differentially expressed mRNAs and protein. Note that mRNAs were measure for all proteins detected but not vice versa. **(C)** Venn diagram showing the overlap in relatively upregulated protein and mRNAs in common. **(D)** Bar graph showing pathway enrichment of MsigDB Hallmark from Enrichr using significant (adjusted *P*-value <0.05) shared upregulated protein and RNA, vertical line indicates adjusted *P*-value of 0.05.

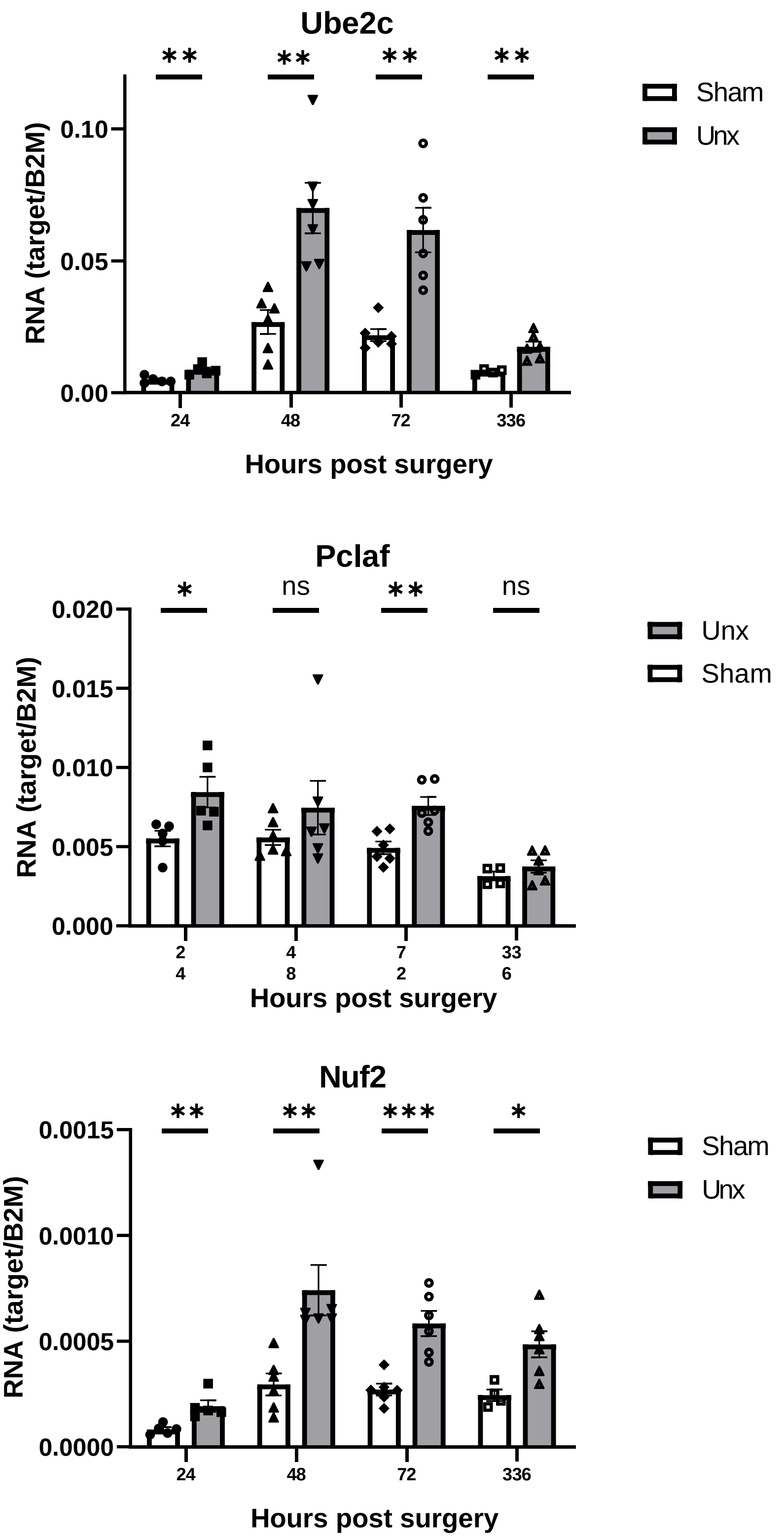

Figure S4: Gene expression changes in the remaining kidney following sham or left unilateral nephrectomy surgery in mice (C57BL6), related to Figure 4. Mice (C57BL6) were subjected to either sham surgery or left unilateral nephrectomy to investigate the molecular response in the remaining kidney to genes involved in cell cycle regulation. At specific time points (24, 48, 72, and 336 hours) after the surgical procedures, the remaining kidney tissue was collected and RNA was extracted. Quantitative PCR (QPCR) analysis was performed to evaluate the relative gene expression levels. The significance levels were denoted as follows: *p < 0.05, **p < 0.01, and ***p < 0.001. Non-significant differences were represented as ns (not significant). The bar graphs illustrate the gene expression ratio normalised to housekeeper gene B2M, and error bars indicate standard error of the mean (SEM). The data are presented as mean ± SEM (n=4-6) for each group.

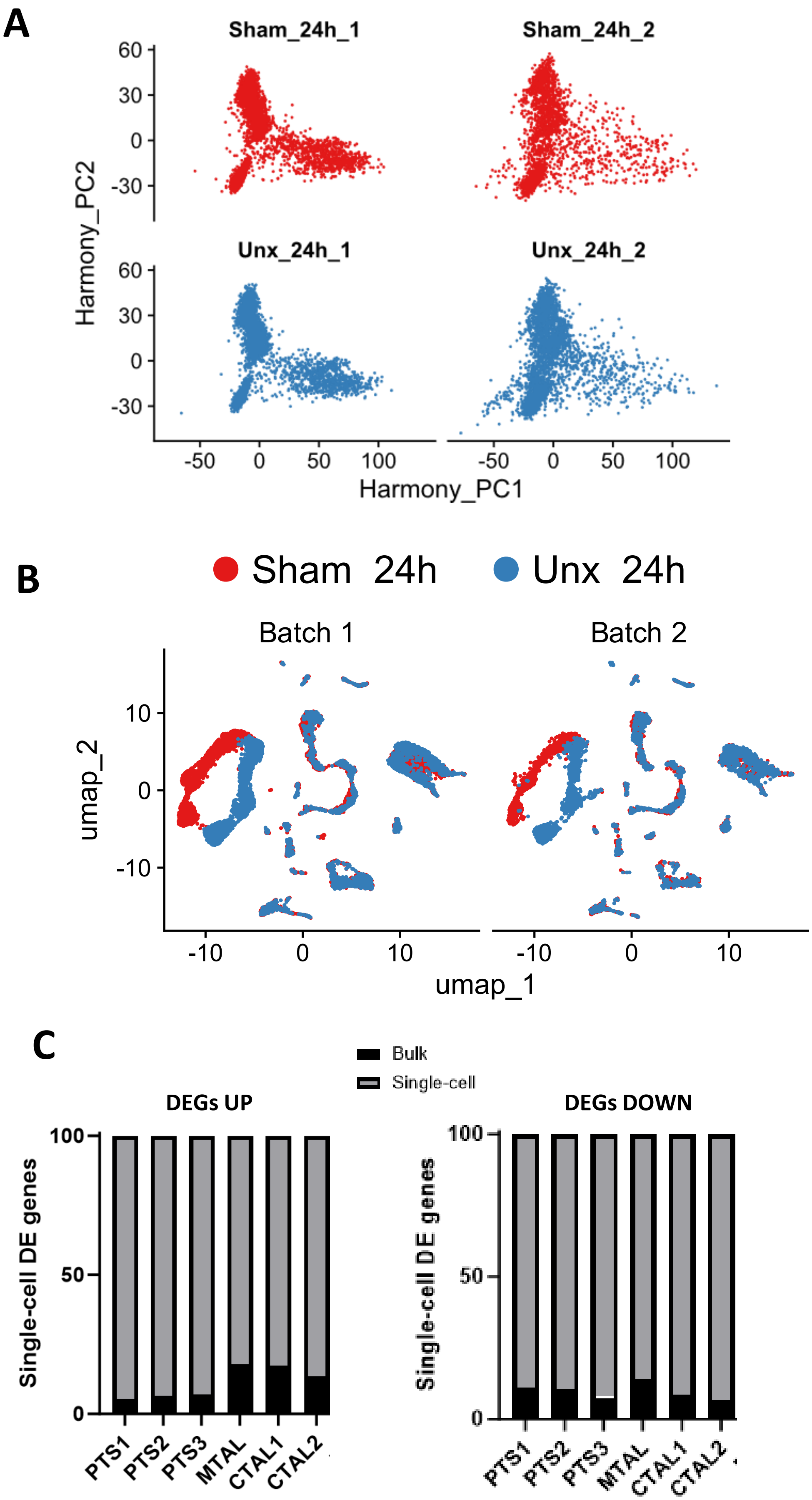

Figure S5: snRNA-seq batch analysis and overlapping gene expression analysis between bulk RNA-seq and snRNA-seq, related to Figure 5. A) PCA of snRNA-SEQ batches 1 and 2 with corrected PCA embeddings using Harmony package in R B) Umap cluster analysis shows comparable clustering of cell types between batches. C) shows percentage overlap of genes upregulated and down regulated in sn-RNA-Seq (in grey) and bulk in black.

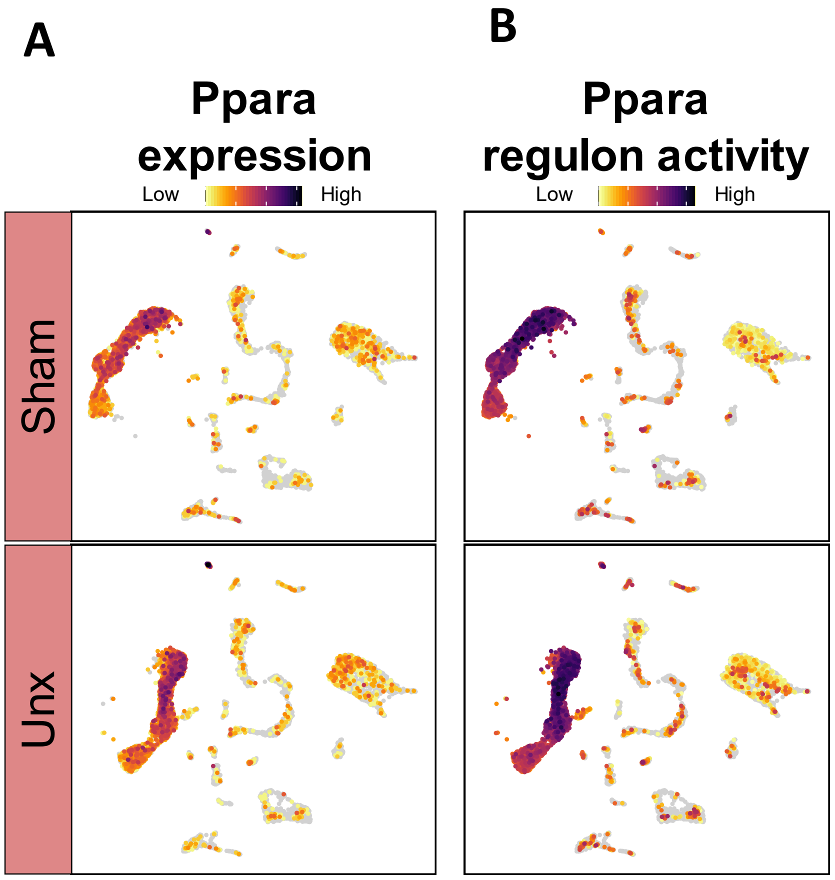

Figure S6: Proximal tubule cells show the highest expression and regulon activity of Ppara without significant changes post nephrectomy, related to Figure 6. UMAP clustering of kidney cells. A) Shows Ppara expression broadcast across all cell types. B) Shows Ppara regulon activity (SCENIC) across all kidney cell types.

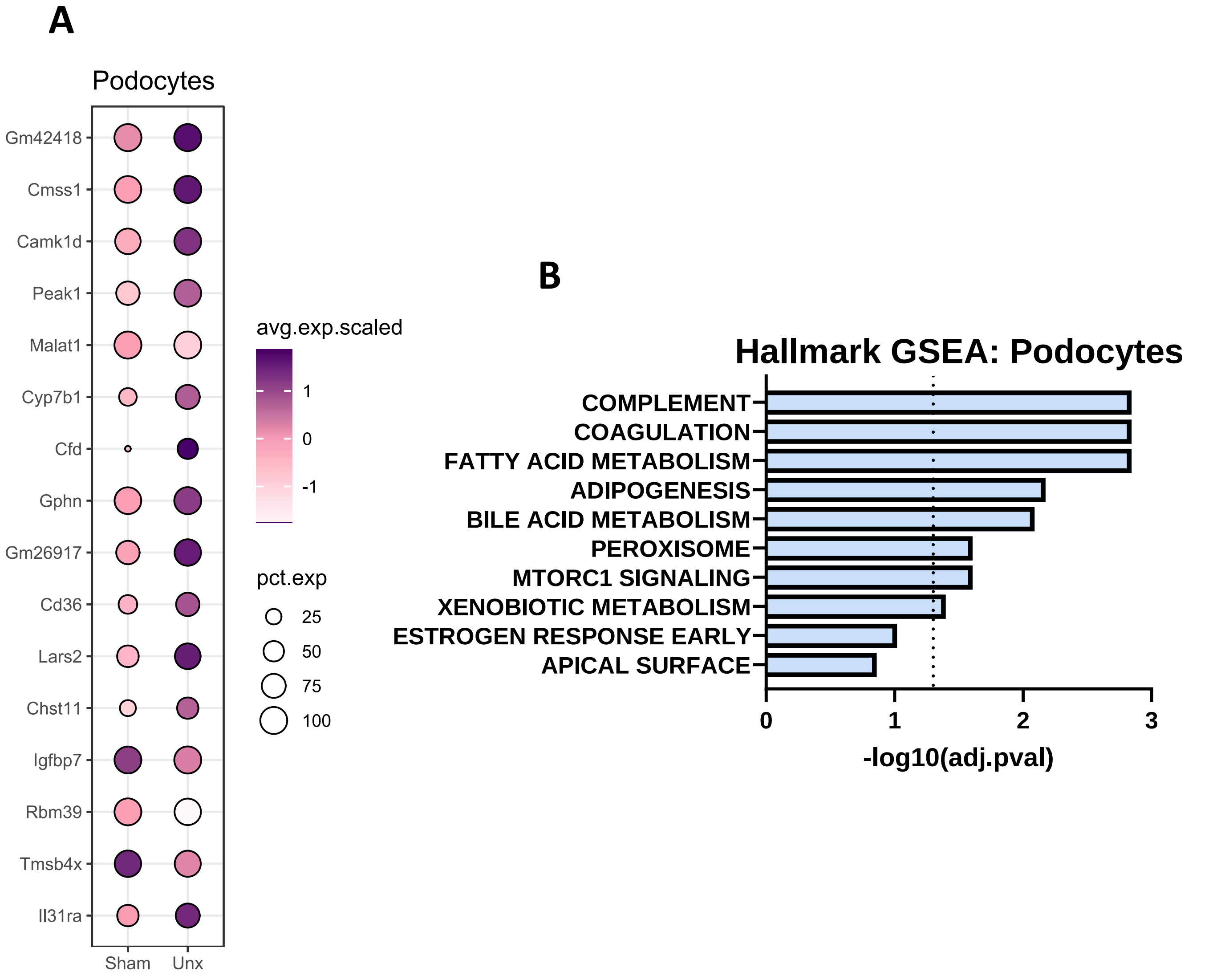

Figure S7: Podocyte differential gene expression hallmark GSEA following nephrectomy, **related to Figure 6.** A) Bubble plot showing all significantly (adjusted P-value <0.05) differentially expressed genes ranked by significance. Colour scale shows average scaled expression and size of bubble shows percentage of cells. B) Genes in podocytes post-unilateral nephrectomy vs. sham operation at 24h, ranked by -log10(p-value) * sign(logFC). Gene set enrichment analysis performed using fgsea package in R with Hallmark gene sets from msigdbr; p-values adjusted by Benjamini-Hochberg method. Barplot of top 10 terms based on -log10(adj. p-value) by , with dotted line indicating adj. p-value = 0.05.

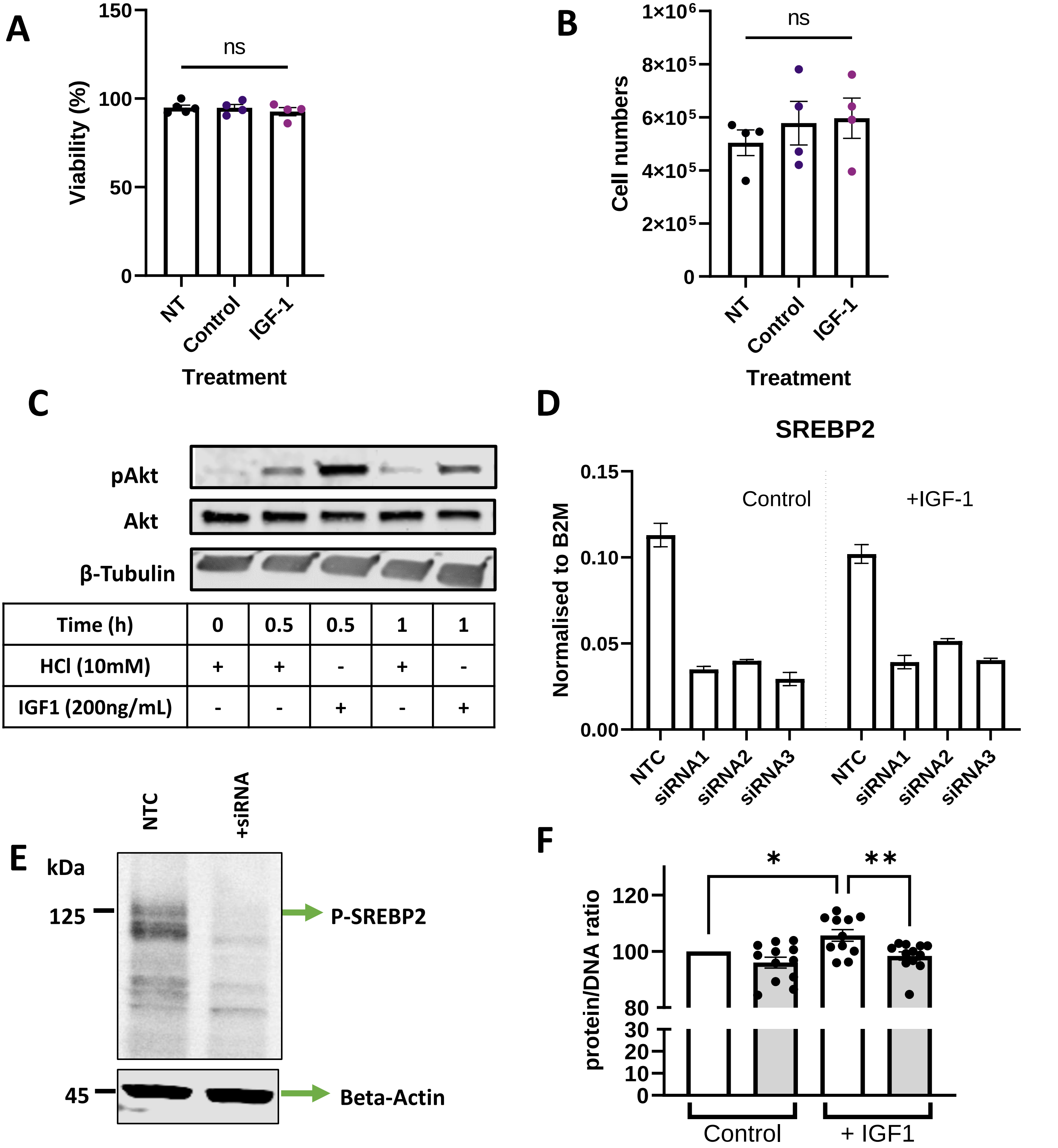

Figure S8: HK2 *in vitro* analysis, related to Figure 7. (A) Cell viability and **(B)** cell numbers of HK2 cells in response to no treatment (NT), control (HCl) and IGF1 (200ng/μ L). Hemocytometer and Trypan Blue staining were used for counting live cells and assessing viability (n=4). **(C)** Serum starved HK2 cells were treated with HCl control or IGF-1 (200ng/mL) for 0, 0.5 or 1 h. Whole cell subjected to SDS-PAGE and then transferred to nitrocellulose membranes. Membranes were probed for pAKT, AKT, and β-tubulin (n=4). **(D)** HK-2 cells were seeded and incubated for 3 days in complete media before transfection with 3 different (1-3) siRNA targeting SREBP2 or NTC control in SFM (serum-free DMEM/F12) for 24h. RNA was then extracted and analysed by qRT-PCR for gene expression of SREBP2 mRNA**.** **(E)** As above HK-2 cells were transfected with siRNA targeting SREBP2 or NTC control in SFM (serum-free DMEM/F12) for 48hours. Whole cell lysates were then prepared and separated on SDS-PAGE and analysed via immunoblot for SREBP2, where the precursor is knocked out with the use siRNA 1 targeting SREBP2. **(F)** HK-2 cells transfected with SREBP2 siRNA or NTC control in SFM for 24h, followed by 48h treatment with/without IGF-1 (200ng/ml). Protein/DNA ratio analysed by flow cytometry; bar graph depicts percentage change vs. control (n=11). Error bars: SEM; one-way ANOVA; *P<0.05, **P<0.01, ****P<0.0001.

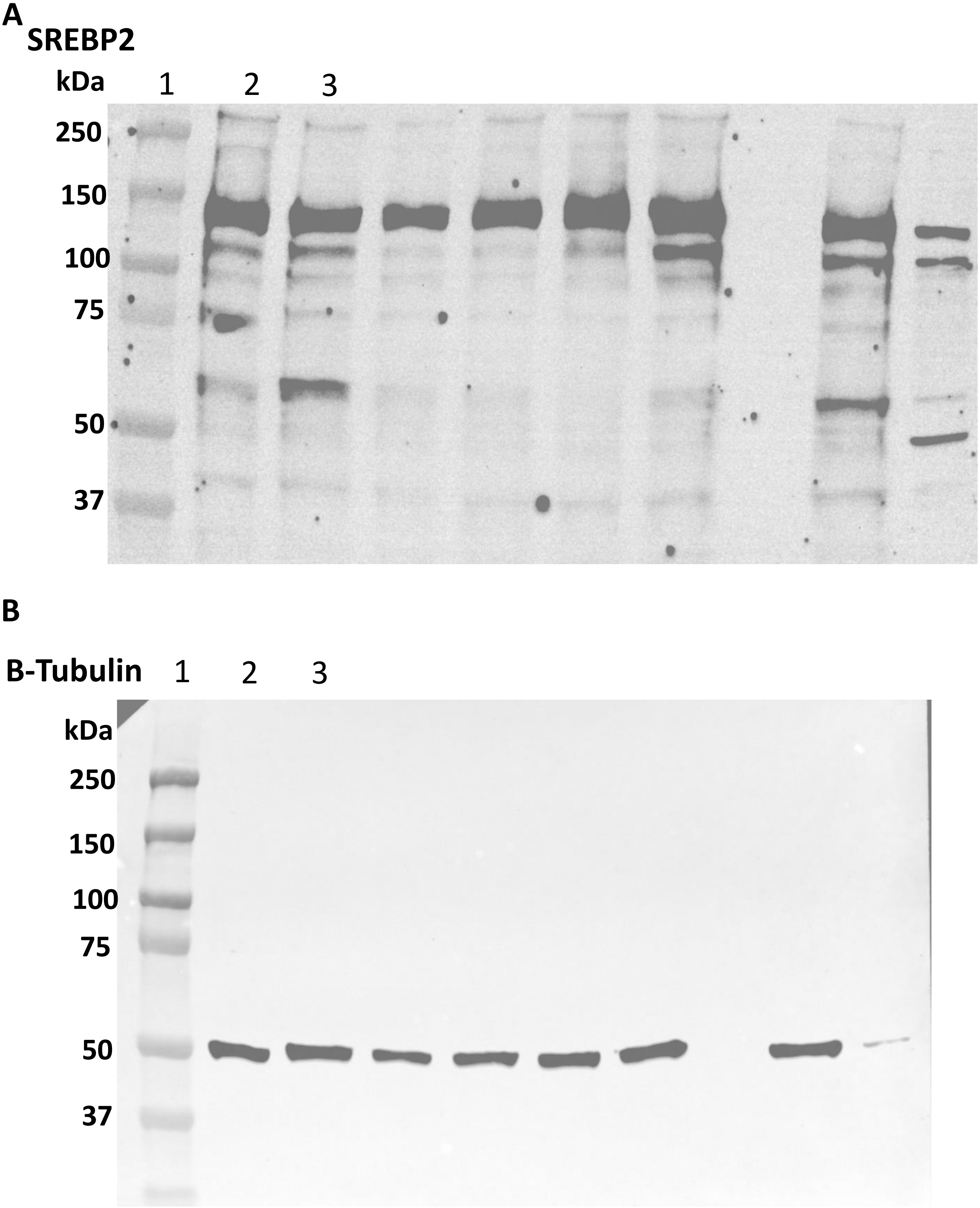

Figure S9: Raw uncropped western blot images from 7F, related to Figure 7. Relevant lanes are 1-3 of each blot which contain the following: Lane 1: Molecular Weight Marker, Lane 2: Control (HCL solution alone), Lane 3: +IGF1(200ng/ml) in HCL solution. **(A)** Immunoblot for SREBP2 **(B)** immunoblot for B-tubulin.

Table S1: Top 20 differentially expressed genes at 24 hours ranked by adjusted *P*-value with a positive log2fold change of ≥ 1, related to Figure 1**.**

Gene Symbol	Description	log2FC	AveExpr	BaseMean	P.Value	adj.P.Val
Ndufb1	NADH:ubiquinone oxidoreductase subunit B1	1.187254279	5.073783748	25.74328152	6.92E-09	3.03E-06
Msmo1	methylsterol monoxygenase 1	1.018720769	5.278627562	27.86390893	8.99E-09	3.70E-06
Tubb2a	tubulin, beta 2A class IIA	1.009325045	5.235267645	27.40802732	1.07E-07	2.12E-05
Aldh1a7	aldehyde dehydrogenase family 1, subfamily A7	1.3462515	3.478171885	12.09767966	2.55E-07	4.10E-05
Lipg	lipase, endothelial	1.808128713	0.765598872	0.586141633	1.99E-06	0.00016127
Slc25a25	solute carrier family 25 (mitochondrial carrier, phosphate carrier), member 25	1.212675284	6.007719722	36.09269626	4.91E-06	0.000300726
Rpl22l1	ribosomal protein L22 like 1	1.122616632	3.069786809	9.423591052	1.16E-05	0.000551996
Nkain1	Na+/K+ transporting ATPase interacting 1	1.09536206	2.92481493	8.554542373	1.23E-05	0.000579018
Dipk1b	divergent protein kinase domain 1B	1.184333852	1.046346586	1.094841178	2.24E-05	0.000879502
Slc16a1	solute carrier family 16 (monocarboxylic acid transporters), member 1	1.021590728	5.20324939	27.07380421	2.27E-05	0.000883725
Asf1b	anti-silencing function 1B histone chaperone	1.670419407	0.089536601	0.008016803	3.68E-05	0.001229074
Rpl10	ribosomal protein L10	1.003369032	5.616399051	31.5439383	8.25E-05	0.002321235
Aurkb	aurora kinase B	1.906682939	0.380011309	0.144408595	9.04E-05	0.002472177
Ttk	Ttk protein kinase	2.271670996	-0.907394509	0.823364796	0.000101046	0.00266446
BC055324	cDNA sequence BC055324	1.232540432	-0.017941089	0.000321883	0.000172162	0.003988219
Gm906	predicted gene 906	1.053152973	3.198988922	10.23353012	0.000173025	0.003994135
Chek1	checkpoint kinase 1	1.238758422	0.070327823	0.004946003	0.000186957	0.004212306
Pcsk9	proprotein convertase subtilisin/kexin type 9	1.337596162	2.843434778	8.085121335	0.000202595	0.004487786
Pappa2	pappalysin 2	1.151685811	0.573828975	0.329279693	0.000465752	0.008337915
P2rx1	purinergic receptor P2X, ligand-gated ion channel, 1	1.022577266	0.676782838	0.458035009	0.000477189	0.008484933

Table S2: Top 20 differentially expressed genes at 24 hours ranked by adjusted *P*-value with a negative log2fold change of ≤-1, related to Figure 1.

Gene Symbol	Description	log2FC	AveExpr	BaseMean	P.Value	adj.P.Val
Hypk	huntingtin interacting protein K	-3.128298561	3.431011093	11.77183712	6.93389E-10	6.51686E-07
Rpl9-ps6	ribosomal protein L9, pseudogene 6	-6.289165243	-1.869927506	3.496628876	2.59221E-09	1.62421E-06
Med31	mediator complex subunit 31	-1.020846437	2.318964813	5.377597805	5.36814E-09	2.72892E-06
Hspb11	heat shock protein family B (small), member 11	-1.101191572	2.350130063	5.523111311	2.14695E-08	6.89014E-06
Abca1	ATP-binding cassette, sub-family A (ABC1), member 1	-1.264601567	4.78649739	22.91055727	3.7394E-07	5.19972E-05
Jph1	junctophilin 1	-1.064491377	0.040353544	0.001628408	2.27009E-05	0.000883725
Ifit1bl2	interferon induced protein with tetratricopeptide repeats 1B like 2	-1.165585179	0.565552347	0.319849457	3.48975E-05	0.001186432
Bhlhe41	basic helix-loop-helix family, member e41	-1.084974839	1.272644079	1.619622952	8.86248E-05	0.002449841
Neil2	nei like 2 (E. coli)	-1.068820119	1.066872165	1.138216216	0.00011008	0.002856866
Rbfox1	RNA binding protein, fox-1 homolog (C. elegans) 1	-1.203017313	0.440588063	0.194117841	0.00012274	0.003082095
E330034L11Rik	RIKEN cDNA E330034L11 gene	-1.225546955	-0.797335029	0.635743149	0.000520291	0.00907957
Derpc	DERPC proline and glycine rich nuclear protein	-2.183828072	-0.195837019	0.038352138	0.000848926	0.013219129
Gm28035	predicted gene, 28035	-1.09737472	1.363180001	1.858259716	0.000857694	0.01328815
Gm43546	predicted gene 43546	-1.403267319	-1.303179684	1.698277289	0.001209035	0.01714276
Tmem158	transmembrane protein 158	-1.3910079	-0.191599625	0.036710416	0.002630669	0.029865694
Pet117	PET117 homolog	-2.860118483	-2.3716166	5.624565299	0.003022447	0.032976253
Gm28539	predicted gene 28539	-4.106144148	-3.267025732	10.67345713	0.00444032	0.043214295

Table S3: Top 20 differentially expressed genes at 48 hours ranked by adjusted *P*-value with a positive log2fold change of ≥ 1, related to Figure 1.

Gene Symbol	Description	log2FC	AveExpr	BaseMean	P.Value	adj.P.Val
Dynlrb1	dynein light chain roadblock-type 1	1.336008482	5.65761675	32.00862729	1.73E-18	2.08E-14
Bola3	bolA-like 3 (E. coli)	1.361741679	5.500473823	30.25521227	4.10E-18	2.08E-14
mt-Nd1	mitochondrially encoded NADH dehydrogenase 1	1.587307147	14.22651239	202.3936548	5.48E-18	2.08E-14
Ndufa12	NADH:ubiquinone oxidoreductase subunit A12	1.274799766	6.774817783	45.89815599	6.33E-18	2.08E-14
Rps26	ribosomal protein S26	1.248677433	6.650313603	44.22667102	1.35E-16	3.56E-13
mt-Nd2	mitochondrially encoded NADH dehydrogenase 2	1.390329549	14.04494079	197.2603617	2.24E-16	4.91E-13
Znhit1	zinc finger, HIT domain containing 1	1.212035287	4.393884011	19.3062167	5.47E-16	1.03E-12
Fxyd2	FXYD domain-containing ion transport regulator 2	1.107055928	10.40129717	108.1869827	9.29E-16	1.53E-12
Hcfc1r1	host cell factor C1 regulator 1 (XPO1-dependent)	1.343271429	5.751869402	33.08400162	3.45E-15	5.04E-12
mt-Nd4	mitochondrially encoded NADH dehydrogenase	1.107826408	14.37379454	206.6059694	4.20E-15	5.53E-12
Cycs	cytochrome c, somatic	1.10240595	7.470541757	55.80899414	5.72E-15	6.84E-12
Nabp2	nucleic acid binding protein 2	1.238161092	5.36454091	28.77829918	1.10E-14	1.21E-11
S100a1	S100 calcium binding protein A1	1.25353934	7.310819433	53.44808078	1.70E-14	1.72E-11
mt-Cytb	mitochondrially encoded cytochrome b	1.144077102	14.8961388	221.8949512	2.90E-14	2.24E-11
Espn	espin [Source:MGI Symbol;Acc:MGI:1861630]	1.132673496	4.794569858	22.98790012	1.73E-13	1.08E-10
mt-Nd5	mitochondrially encoded NADH dehydrogenase	1.254845527	14.27369571	203.7383891	4.79E-13	2.03E-10
Tomm6	translocase of outer mitochondrial membrane 6	1.224299874	5.686321031	32.33424687	5.15E-13	2.03E-10
Rpl28	ribosomal protein L28	1.134577626	6.746298896	45.51254879	5.26E-13	2.03E-10
Chchd10	coiled-coil-helix-coiled-coil-helix domain containing 10	1.060058479	8.08653779	65.39209343	5.55E-13	2.09E-10
mt-Atp8	mitochondrially encoded ATP synthase 8	1.25350775	11.85167968	140.4623113	1.35E-12	4.24E-10

Table S4: Top 20 differentially expressed genes at 48 hours ranked by adjusted *P*-value with a negative log2fold change of ≤-1, related to Figure 1.

Gene Symbol	Description	log2FC	AveExpr	BaseMean	P.Value	adj.P.Val
Rpl22l1	ribosomal protein L22 like 1	-1.993732786	3.069786809	9.423591052	3.44E-13	1.70E-10
Rpl10	ribosomal protein L10	-2.145072013	5.616399051	31.5439383	4.98E-13	2.03E-10
Ndufb1	NADH:ubiquinone oxidoreductase subunit B1	-1.479440034	5.073783748	25.74328152	5.08E-12	1.31E-09
Rpl39	ribosomal protein L39	-1.113770017	5.411417209	29.28343621	1.77E-10	2.03E-08
Rps6	ribosomal protein S6	-1.015390575	7.13942614	50.97140561	1.79E-10	2.03E-08
Zfp97	zinc finger protein 97	-1.376454185	1.836498244	3.372725802	4.25E-10	3.83E-08
Spink1	serine peptidase inhibitor, Kazal type 1	-1.140461095	7.661063116	58.69188806	4.47E-10	3.89E-08
Snrpg	small nuclear ribonucleoprotein polypeptide G	-1.091923505	2.711939717	7.354617027	6.46E-09	3.15E-07
Smim26	small integral membrane protein 26	-1.137179872	2.392547045	5.724281361	2.82E-08	1.09E-06
Ahnak	AHNAK nucleoprotein (desmoyokin)	-1.052566239	6.348558761	40.30419834	2.98E-08	1.14E-06
AU041133	expressed sequence AU041133	-1.257349164	1.14772028	1.317261841	3.82E-08	1.37E-06
Tspan4	tetraspanin 4	-1.023253106	4.233795404	17.92502352	8.57E-07	1.83E-05
Zfp965	zinc finger protein 965	-1.115868207	1.64655684	2.711149428	1.05E-06	2.14E-05
Zfp966	zinc finger protein 966	-1.119412578	2.327977521	5.419479337	1.29E-06	2.53E-05
Cfh	complement component factor h	-1.168090265	6.881644295	47.3570282	1.59E-06	3.02E-05
Styx	serine/threonine/tyrosine interaction protein	-1.138413334	2.710278327	7.345608608	2.24E-06	4.06E-05
Dynlt1c	dynein light chain Tctex-type 1C	-1.504864503	0.587210329	0.344815971	2.52E-06	4.46E-05
H2-Ob	histocompatibility 2, O region beta locus	-1.724256141	-0.507445763	0.257501202	3.32E-06	5.64E-05
Bok	BCL2-related ovarian killer	-1.146431478	3.191931238	10.18842503	5.26E-06	8.37E-05
C7	complement component 7	-1.467602563	2.336328545	5.458431068	6.41E-06	9.85E-05

Table S5: Top 20 differentially expressed genes at 72 hours ranked by adjusted *P*-value with a positive log2fold change of ≥ 1, related to Figure 1.

Gene Symbol	Description	log2FC	AveExpr	BaseMean	P.Value	adj.P.Val
Ttr	transthyretin	1.415770611	6.485231486	42.05822743	5.25E-08	0.000690906
Ckap2	cytoskeleton associated protein 2	1.489074711	0.56723626	0.321756974	1.20E-07	0.000789076
Slc25a25	solute carrier family 25 (mitochondrial carrier, phosphate carrier), member 25	1.360478566	6.007719722	36.09269626	2.79E-07	0.00095858
Anln	anillin, actin binding protein	1.1746276	1.642455823	2.697661129	3.77E-07	0.00095858
Kif20a	kinesin family member 20A	1.21947039	2.138568737	4.573476241	5.45E-07	0.001024047
Enox1	ecto-NOX disulfide-thiol exchanger 1	1.463977942	0.813408699	0.661633711	8.40E-07	0.001206232
Nat8f5	N-acetyltransferase 8 (GCN5-related) family member 5	2.008030846	4.023423888	16.18793978	1.00E-06	0.001206232
Pck1	phosphoenolpyruvate carboxykinase 1, cytosolic	1.062956666	10.91349035	119.1042716	1.01E-06	0.001206232
Tk1	thymidine kinase 1	1.18423507	2.712459446	7.357436245	1.36E-06	0.001306296
Ube2c	ubiquitin-conjugating enzyme E2C	1.478125662	0.749975487	0.562463231	1.39E-06	0.001306296
Slc16a1	solute carrier family 16 (monocarboxylic acid transporters), member 1	1.027976016	5.20324939	27.07380421	1.83E-06	0.00131259
Cdc20	cell division cycle 20	1.504514661	1.630874334	2.659751094	1.87E-06	0.00131259
Ccnb1	cyclin B1	1.333859893	0.872883025	0.761924775	1.91E-06	0.00131259
Ccnb2	cyclin B2	1.377626624	1.09107296	1.190440204	2.29E-06	0.001366692
Cenpf	centromere protein F	1.025287681	1.696916916	2.879527018	2.50E-06	0.001430096
Ccna2	cyclin A2	1.211830816	2.378423664	5.656899127	3.06E-06	0.001680177
Cdk1	cyclin-dependent kinase 1	1.245039462	1.649281568	2.720129689	3.34E-06	0.001759788
Prc1	protein regulator of cytokinesis 1	1.21669389	1.949823779	3.80181277	3.79E-06	0.001911276
Top2a	topoisomerase (DNA) II alpha	1.080701249	3.085469768	9.520123689	4.95E-06	0.002245787
Ckap2l	cytoskeleton associated protein 2-like	1.273574998	1.390395512	1.93319968	5.59E-06	0.002450115

Table S6: Top differentially expressed genes at 72 hours ranked by adjusted *P*-value with a negative log2fold change of ≤-1, related to Figure 1.

Gene Symbol	Description	log2FC	AveExpr	BaseMean	P.Value	adj.P.Val
Dbp	D site albumin promoter binding protein	-2.104770825	5.2266276	27.31763607	4.37109E-07	0.00095858
Per3	period circadian clock 3	-1.024353602	3.994148338	15.95322095	1.13891E-06	0.001248815
Il5ra	interleukin 5 receptor, alpha	-1.36500414	1.263352821	1.59606035	2.24256E-05	0.006942289
Cyp24a1	cytochrome P450, family 24, subfamily a, polypeptide 1	-2.217861826	5.677864017	32.23813979	6.25948E-05	0.013284235
Bhlhe41	basic helix-loop-helix family, member e41	-1.293962972	1.272644079	1.619622952	9.4628E-05	0.018310513
Ypel1	yippee like 1	-1.029976163	-0.618109793	0.382059716	0.000187614	0.026954246
Cyp26b1	cytochrome P450, family 26, subfamily b, polypeptide 1	-2.278843051	2.583583447	6.67490343	0.000282278	0.033559367
Stra6	stimulated by retinoic acid gene 6	-1.622175432	1.321301466	1.745837564	0.000456934	0.043567679
Sema5b	sema domain, seven thrombospondin repeats (type 1 and type 1-like), transmembrane domain (TM) and short cytoplasmic domain, (semaphorin) 5B	-1.826715405	-0.425305403	0.180884686	0.000494188	0.044948812

Table S7: Additional Key Resource Oligonucleotides, related to Star Methods.

Oligonucleotides		
Nuf2 (Mm01283863_m1)	Life Technologies	Cat# 4331182
Pclaf (Mm00783087_s1)	Life Technologies	Cat# 4331182
Ube2c (Mm00835439_g1)	Life Technologies	Cat# 4331182
Primer: SREBP2 Forward:<br><br>TGTGTATGTCCTGTGCCTTTTC	*Kondo et al.*[67]	N/A
Primer: SREBP2 Reverse:<br><br>TGGGACACAGTGACTGATTGAT	*Kondo et al.*[67]	N/A
Primer: HMGCS1 Forward:<br><br>TTGCTCTATTTGCTGACATGCT	*Kondo et al.*[67]	N/A
Primer: HMGCS1 Reverse:<br><br>TGGCCAAAGAGGTATGAAGTTT	*Kondo et al.*[67]	N/A
Primer MSMO1 Forward:<br><br>ATCATGAGTTTCAGGCTCCATT	*Kondo et al.*[67]	N/A
Primer: MSMO1 Reverse:<br><br>AAGCACGATTCCAATGAAAAAT	*Kondo et al.*[67]	N/A
